# Haplotype-resolved genome assembly provides insights into the unique floral scent of *Rosa rugosa* originated in China

**DOI:** 10.1186/s43897-025-00210-x

**Published:** 2026-04-07

**Authors:** Xi Cheng, Xin Geng, Dan Gao, Hongli Wang, Guoliang Wang, Dongliang Chen, Kang Gao, Tianyi Wang, Chengzhi Jiao, Beibei Jiang, Conglin Huang, Fei Shen

**Affiliations:** 1 Institute of Grassland, Flowers and Ecology, Beijing Academy of Agriculture and Forestry Sciences, Beijing, 100097 China; 2 Beijing Key Laboratory of Agricultural Genetic Resources and Biotechnology, Institute of Biotechnology, Beijing Academy of Agriculture and Forestry Sciences, Beijing, 100097 China; 3 College of Landscape Architecture, Sichuan Agricultural University, Chengdu, 611130 China; 4 Smartgenomics Technology Institute, Tianjin, 300457 China

**Keywords:** Haplotype genome, *R. rugosa*, Floral scent, 2-phenylethanol, RrPrAO

## Abstract

*Rosa rugosa* is an important aromatic plant and produces flowers that are used in medicine, food, plant essential oils, hydrosols, and other aromatic products. 2-phenylethanol (2-PE) is the main effective substance in the volatile organic compounds of rose fragrance, and its synthesis mechanism in *R. rugosa* needs to be investigated.

We herein present a haplotype-resolved genome of *R. rugosa* cv. Hanxiang (HX). Pathways mediating the synthesis of scent-related metabolites were deciphered in depth. Allelic imbalances reveal the distinct roles of different haplotyping in shaping the key trait including fragrance during the flower development stages.

We deciphered the population structure and genetic composition of rose. Selected and mutated genes in groups of separated aromas including but not limited to one rate-limiting enzymes, primary amine oxidases, is responsible for the high level of 2-PE biosynthesis in *R. rugosa*, which could provide new genetic resources for enhancing aroma in other species.

**Supplementary Information:**

The online version contains supplementary material available at 10.1186/s43897-025-00210-x.

## Core

Roses have long been admired for their fragrance, yet the genetic determinants of this trait remain unclear. Using a haplotype-resolved genome of *R. rugosa*, we reveal how allelic diversity and gene introgression shape floral scent metabolism. The discovery of a primary amine oxidase mediated 2-PE biosynthetic pathway uncovers a previously unrecognized mechanism of rose aroma evolution and provides valuable genetic targets for enhancing fragrance in modern breeding.

## Gene and accession numbers

The genome sequencing data, genome assembly, and annotation have been deposited at the National Genomics Data Center (NGDC, https://www.cncb.ac.cn/) under accession number PRJCA016023.

## Introduction

*R. rugosa*, a deciduous shrub belonging to the family Rosaceae, originated in China and other regions in Asia (e.g., Japan). It is a commonly cultivated flower species with characteristics that make it useful in many industries and produces flowers that are used in medicine, food, plant essential oils, hydrosols, and other aromatic products. *R. rugosa*, together with other species of the rose genus, are representative materials for the study of floral volatile organic compounds (China 2014; Commission 2005; Committee, 2015). According to the Chinese Pharmacopoeia, the Dictionary of Traditional Chinese Medicine and the Compendium of Collecting the Lost Works (China 2014; Commission 2005), *R. rugosa* is a rich source of amino acids and trace elements needed by humans and may be useful for treating endocrine disorders and promoting blood circulation (Committee, 2015). Its petals contain relatively high amounts of terpenes, phenols, esters, and alcohols, including phenylethanol, citronellol, geraniol, linalool, and eugenol, which have neuroprotective (Orhan et al. 2009), antioxidative (Hancianu et al. 2013; Senol et al. 2013), anxiolytic (Linck et al. 2010), anti-inflammatory (Wang et al. 2021), and skin-protective (Gunaseelan et al. 2016) effects. The combination of these volatile organic compounds makes up the characteristic rose scent bouquet. Phenylpropanoid-related compounds such as 2-phenylethanol (2-PE) have been shown to contribute greatly to the rose scent, and the amount of 2-PE content dominated the aroma type of rose germplasm (Roccia et al. 2019). 2-PE is widely used in cosmetic flavors and is also used as a substrate for the synthesis of other flavors or pharmaceutical compounds, such as phenylacetic acid, phenylacetaldehyde and phenethyl acetate (Białecka-Florjańczyk et al. 2012; Çelik et al. 2004), and has anticholinesterase activity, which can play a neuroprotective role (Gurdal Orhan, 2009; Senol et al. 2013).

L-phenylalanine (Phe) is a direct precursor of 2-PE and *β*-D-glucopyranoside (2-PEG). The biosynthesis of phenylpropanoid compounds in the flowers of *R. spp.* is mainly conducted in two ways. First, Phe is converted to phenylacetaldehyde (PAA) through aromatic amino acid decarboxylase (AADC) and phenylacetaldehyde synthase (PAAS). PAA was then converted to 2-PE by phenylacetaldehyde reductase (PAR) (Chen et al. 2011). Second, aromatic amino acid aminotransferase (AAAT) catalyzed L-Phe to produce phenylpyruvic acid (PPA), and RNAi inhibition of AAAT gene RyAAAT3 decreased 2-PE production in *R. hybrida* protoplasts (Hirata et al. 2016). However, the mechanism of 2-PE synthesis in *R. rugosa* was unclear and needed to be investigated.

The current *R. rugosa* reference genome (454.78 Mb), which was assembled using PacBio circular consensus sequencing (CCS) data, is from a single-petal, self-compatible and fruit-bearing wild germplasm (hereafter referred to as PRr) (Chen et al. 2021) that has been widely used as stock. *R. rugosa* cv. Plena, which has the characteristics of rich aroma and high double petals, are regarded as the most influential germplasm in the history of global scented rose breeding, and yet the absence of the genome assembly of this fundamental material has hindered full exploration of the genetic basis of *R. rugosa* improvement.

Because of its highly heterozygous genome, *R. rugosa* cv. Plena is a valuable species for investigating allelic variations with potentially important effects during evolution. Hybridizations involving various *R. rugosa* cultivars have generated offspring with desirable traits superior to those of either parent; this heterosis has been exploited in rose breeding programs. In this study, we assembled a chromosome-scale genome of *R. rugosa* cv. Hanxiang (HX), with two haplotypes fully represented. Core genes in the aroma synthesis pathway were characterized by a weighted gene co-expression network analysis (WGCNA) and the allelic imbalances analysis. Our results provide insights into the mechanism underlying heterosis of *R. rugosa* and the formation of rose floral scents.

## Results

### Genome assembly and annotation

Based on *k*-mer analysis (Fig. S1A, Table S1) using 25.93G Illumina reads, the HX genome size was estimated to be approximately 456.19 Mb, with a heterozygosity level of 1.52%, which was consistent with the genome size revealed by the flow cytometry analysis (approximately 420 Mb) (Fig. S1B, Table S2).

**Fig. 1:**
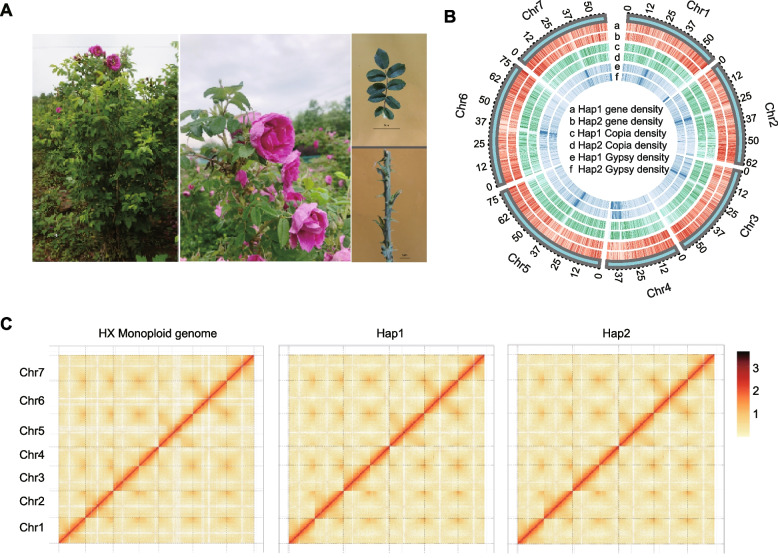
Assembly, composition, and evolution of the HX genome. **A** Images of *R. rugosa* HX plant parts. Scale bar, 5 cm (upper panel) and 1 cm (lower panel). **B** *R. rugosa* HX genomic features presented in a Circos diagram of hap1 and hap2. The circles (outer to inner) indicate gene density (a: hap1, b: hap2), Copia density (c: hap1, d: hap2), and Gypsy density (e: hap1, d: hap2). **C** Contact map of the Hi-C links among seven pseudochromosomes

On the basis of PacBio HiFi data and Hi-C data (Table 1), the hifiasm software generated three assemblies, primary contigs (monoploid assembly), and fully phased contigs of two haplomes (hap1 and hap2). The three assemblies, with a total length that was close to the estimated genome size, were clustered into seven pseudochromosomes using high-throughput chromatin conformation capture (Hi-C) data (125 × coverage) (Table S3, Fig. 1C, Table S4, Fig. S2). To confirm the chromosomal basis for the seven pseudochromosomes, we performed cytological karyotype analysis using mitotic cells from leaf buds of HX. The results confirmed that HX is diploid with 2n = 2x = 14 chromosomes, supporting the assembly of seven pseudochromosomes. Chromosomes were classified into 10 metacentric and 4 submetacentric types (karyotype formula: 10 m + 4sm) (Fig. S3). The monoploid, hap1, and hap2 assemblies were 97.7%, 98.9%, and 99.0% complete according to the Benchmarking Universal Single-Copy Orthologs (BUSCO v.7.1) analysis (Fig. S3), which are higher than the PRr genomes (93.2%) (Table S5) (Chen et al. 2021). The LTR Assembly Index (LAI) values of the three assemblies exceeded 20 (i.e., reference level) (Fig. S4). Whole-genome comparisons showed a high degree of synteny between PRr and our three assemblies. These results reflect the high accuracy and completeness of *R. rugosa* genome assembly (Fig. S5, S6).

**Table 1:** Details regarding the *R. rugosa* HX genome assembly and annotation

Assembly characteristics	Hap1	Hap2	Monoploid
Total length of contigs	453.62 M	440.52 M	468.87 M
N50 length of contigs	7.98 M	11.01 M	24.41 M
Total number of contigs	291	177	183
Longest contigs	16.45 M	25.74 M	54.40 M
Total length of scaffolds	453.63 M	440.53 M	468.88 M
N50 length of scaffolds	60.56 M	58.78 M	59.40 M
Total number of scaffolds	172	99	154
Longest scaffolds	80.14 M	76.04 M	75.35 M
Total gap size	11.90 K	7.80 K	2.90 K
Total sequences anchored to the pseudochromosomes	443.16 M	434.14 M	431.47 M
Number of annotated high-confidence genes	36,141	35,781	36,023
Percentage of transposon element sequences	50.38%	51.37%	51.80%
Complete BUSCOs	98.50%	98.60%	97.40%
Fragmented BUSCOs	0.70%	0.60%	0.70%
Missed BUSCOs	0.80%	0.80%	1.90%
LAI	21.91	21.4	21.13

By integrating de novo, homologous (*R. chinensis*, *R. multiflora*, *R. rugosa*, *Fragaria vesca*, *Malus domestica*, and *Prunus persica*), and transcript (roots, stems, leaves, petals, stamens, pistils, sepals, and petioles) predictions, 36,023, 36,141, and 35,781 genes were predicted in the HX monoploid, hap1, and hap2 assemblies, respectively (Tables S6, S7, S8, Fig. S7). At least 96% of the genes in all three genomes have functional annotation (Table S9). Transposable elements rates are around 50% (Table S10). Non-coding RNA sequences were also annotated (Tables S11, S12, S13).

We compared the syntenic patterns of the HX monoploid genome with those of the PRr and *R. chinensis* (Rchi) genomes (Fig. S8). We find that HX had highly conserved syntenic relationships with PRr and Rchi, with closely matched chromosomes. Large structural variants (SVs) were detected among the three genomes (Fig. S8; green blocks)*.* Chromosomes 1, 3, and 7 of the HX monoploid genome were similar to Rchi chromosomes 4, 1, and 7, respectively*.* However, compared with chromosome 1 of PRr, the head of chromosome 1 of the HX heterozygous genome had large inversions. Moreover, two large SVs were detected between chromosome 7 of the HX monoploid genome and PRr chromosome 7 (Table S14).

### Expansion gene and expansion model analysis

**Fig. 2:**
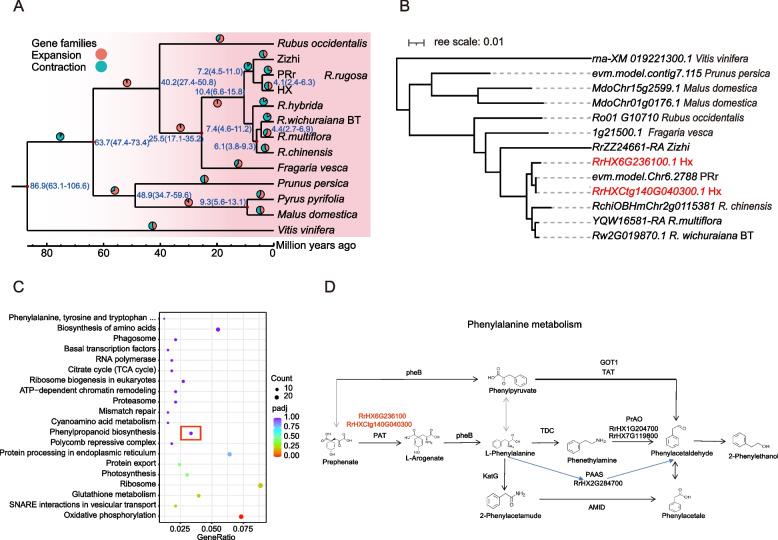
Expansion gene and expansion model. **A** Evolutionary tree, divergence time and expansion and contraction results. **B** Phylogenetic tree of expanded genes. **C** KEGG pathway which expanded genes enriched. **D** Expanded genes in phenylalanine synthesis pathway

We constructed an evolutionary tree (Fig. 2A) using 854 single-copy genes from 13 species (HX, PRr (Chen et al. 2021), *R. wichuraiana* ‘Basye’s Thornless’ (BT) (Zhong et al. 2021), *R. chinensis* (Hibrand Saint-Oyant et al. 2018), *Rosa hybrida* (Zhang et al. 2024), *Rosa multiflora*, Zizhi (Shang et al. 2024), *Prunus persica*, *Fragaria vesca*, *Malus domestica*, *Pyrus pyrifolia*, *Rubus occidentalis*, *Vitis vinifera* (downloaded from https://www.rosaceae.org/)). It was found that HX and PRr differentiated at ~ 4.5 millon years ago (Mya). *R. chinensis* and BT differentiated 6.3 Mya. HX have 1,599 expansion and 1,105 contraction gene families. The significantly expanded gene family is mainly enriched in Glutathione metabolism, Photosynthesis, Phenylpropanoid biosynthesis, Phenylalanine, tyrosine and tryptophan biosynthesis KEGG pathways (Fig. 2C). We focused on phenylalanine synthesis pathway which is related to the synthesis of floral substances 2-PE, and in this pathway there were expansion genes *RrHX6G236100.1* and *RrHXCtg140G040300.1* which encoded bifunctional aspartate aminotransferase and glutamate (PAT) and belonged PAT gene family (Fig. 2B, D).

### Haplotype variations and allelic imbalance

**Fig. 3:**
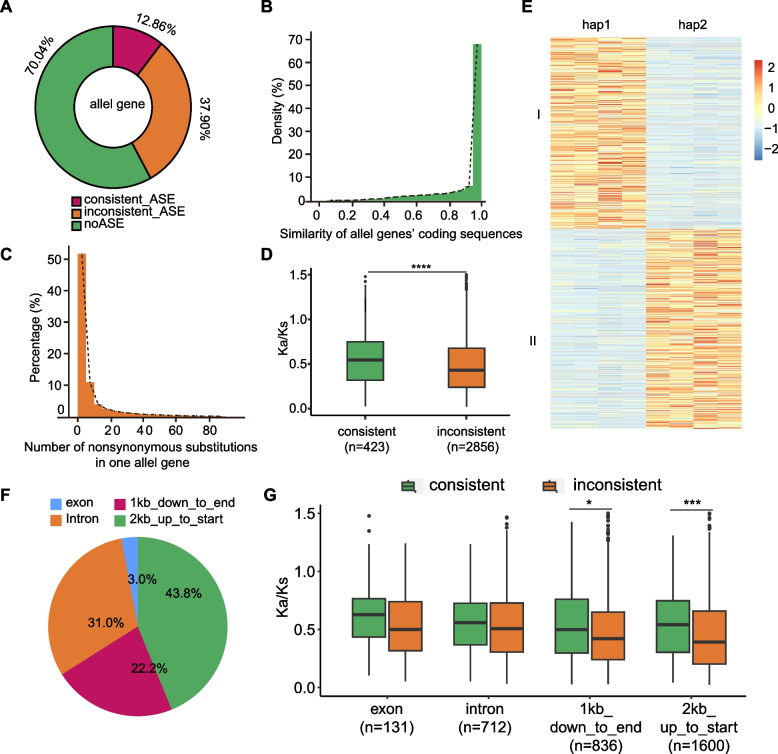
Genetic variations between haplotypes and allelic imbalance in *R. rugosa*. **A** Allele identification. A total of 21,033 alleles were identified in hap1 and hap2, of which 718 were consistent (12.86%), 5583 were inconsistent (37.90%), and 14,732 were noASE (70.04%). **B** Similarities among allele coding sequences. **C** Distribution of the number of non-synonymous substitutions between alleles. **D** Identification of ASE gene selection pressure by ka/ks value between consistent and inconsistent ASE. Consistent ASE is significantly higher than inconsistent, indicating that consistent ASE is under greater selection pressure. **E** Transcriptome expression heatmap of consistent ASE in four stages. The horizontal axis represents four transcriptomic periods at a time, and genes can be divided into two classes, class I are hap1’ consistent ASEs which have higher FPKM in hap1 in the four stages. Class II are hap2’ consistent ASEs. **F** SV distribution on ASE gene. 36,307 SV were detected betweent hap1 and hap2. These SV are mainly distributed in the promoter(2 Kb up to start codon). **G** Selection pressure of different gene regions. Consistent ASE was significantly higher than non-consistent ASE in both upstream 2 K and downstream 1 K genes

On the basis of the phased haplotypes, we separated 21,033 gene pairs (56.37%) as alleles. The coding sequences of most allelic genes were highly similar (median = 0.99; Fig. 3B). 83.96% of the allelic genes contained at least one non-synonymous substitution that may have influenced protein functions (Fig. 3C). 81.92% of the allelic genes underwent purifying selection (Ka/Ks ratio < 1) (Fig. S9A).

Petal transcriptome data for HX were generated at four flower development stages (bud stage, initial opening period, bloom period, and fading period) to explore imbalanced allele expression. Among 21,033 alleles identified, 718 pairs (12.86%) of consistent ASE genes were obtained (Fig. 3A, Table S15). Consistent ASE means that the expression of one haplotype was significantly higher than that of another haplotype in all four periods (Fig. 3E). 5,583 pairs (37.90%) of inconsistent ASE were obtained. The rest were non-ASE genes, 14,732 pairs (79.04%). To assess possible natural selection on allelic gene expression, we calculated Ka and Ks values between consistent ASE and consistent ASE (Fig. 3D). Consistent ASE had significantly higher Ka/Ks than inconsistent ASE (t-test, *P* value = 9.6 × 10^−6^), indicating that Consistent ASE were under greater positive selection pressure (Fig. 3C). Of these, 280 alleles showed possible positive selection (Ka/Ks > 1).

Based on the variation detection results between the two haplotypes, we analysized the relationship between ASE gene and SV. The majority of SV (43.8%) was found to be concentrated in the promoter region of the ASE gene (2 kb up to start codon) (Fig. 3F). About 31.0% and 22.2% SVs were located in intron and down to end 1 kb. The lowest proportion (3.0%) was found in exon, indicating the conserved nature between ASE genes. In the promoter region and downstream of the gene, the consistent ASE was experienced to significant positive selection pressure (Fig. 3G). We performed transcription factor prediction on SV-affected ASE genes, the ASE gene transcription factors in promoter region were correlated with MYB, B3 and ERF (Table S16).

The consistent and inconsistent allelic expression patterns suggested that allele functions may vary among flower development stages because of different regulatory pathways (Fig. S9). We compared the hap1 and hap2 gene expression levels in stage 3 (i.e., most important flower development stage) at the chromosome level using sliding windows (Fig. S9C). There were 21 significantly enriched biological pathways among the alleles specifically expressed in HX (stage 3), including glycerophospholipid metabolism, terpenoid backbone biosynthesis, ascorbate and aldarate metabolism, and inositol phosphate metabolism, which is related to biosynthesis of monoterpenoids (such as citronellol et al.) and phenylpropanoids (such as 2-phenylethanol et al.) that has been shown to contribute greatly to the typical scents of *R. rugosa* and other *Rosa* species (Tables S17, S18).

Genes in phenylalanine synthesis pathway reflected different modes of expression (Table S19). The expression patterns of *RrHX2G284700* encoding PAAS differed between hap1 and hap2. The genomes of these two assembly haplotypes were used as the reference material for investigating the asymmetrical expression of the screened alleles (Fig. 3D). PAAS (*RrHX2G284700*) of HX had same allelic expression patterns in different periods, manifesting a consistent ASE gene and the dominance effect. However, *RrHX6G236100.1* belong to PAT, the primary amine oxidase (PrAO)-encoding genes *RrHX1G204700* and *RrHX7G119800* were no ASE gene between hap1 and hap2. The content of 2-PE in HX reaches high levels across various floral stages, with measurements of 1.39 μg/g during the bud stage, 1.88 μg/g at the initial opening period, 8.64 μg/g at bloom period, and 7.42 μg/g during the fading period. In contrast, Guomeigui (GM) (*R*. *rugosa* Thunb. f. *rosea* Rehd.) characterized by a haplopetalous corolla structure with five petals and capable of producing fruit (Fig. S10), exhibits significantly lower levels of 2-PE in the petals. During the bloom stage, 2-PE is nearly undetectable in GM, with concentrations of 0.16 μg/g in the bud stage, 0.04 μg/g at initial opening, and 0.12 μg/g at the fading period. Compared with HX, GM had more consistent ASE genes (Table S15). Therefore, we speculate that the differential expression of alleles is one of the reasons for the different flavor types of the two *R. rugosa* plants with highly heterozygous and relatively homozygous.

### Identification of conserved and/or divergent gene co-expression modules

**Fig. 4:**
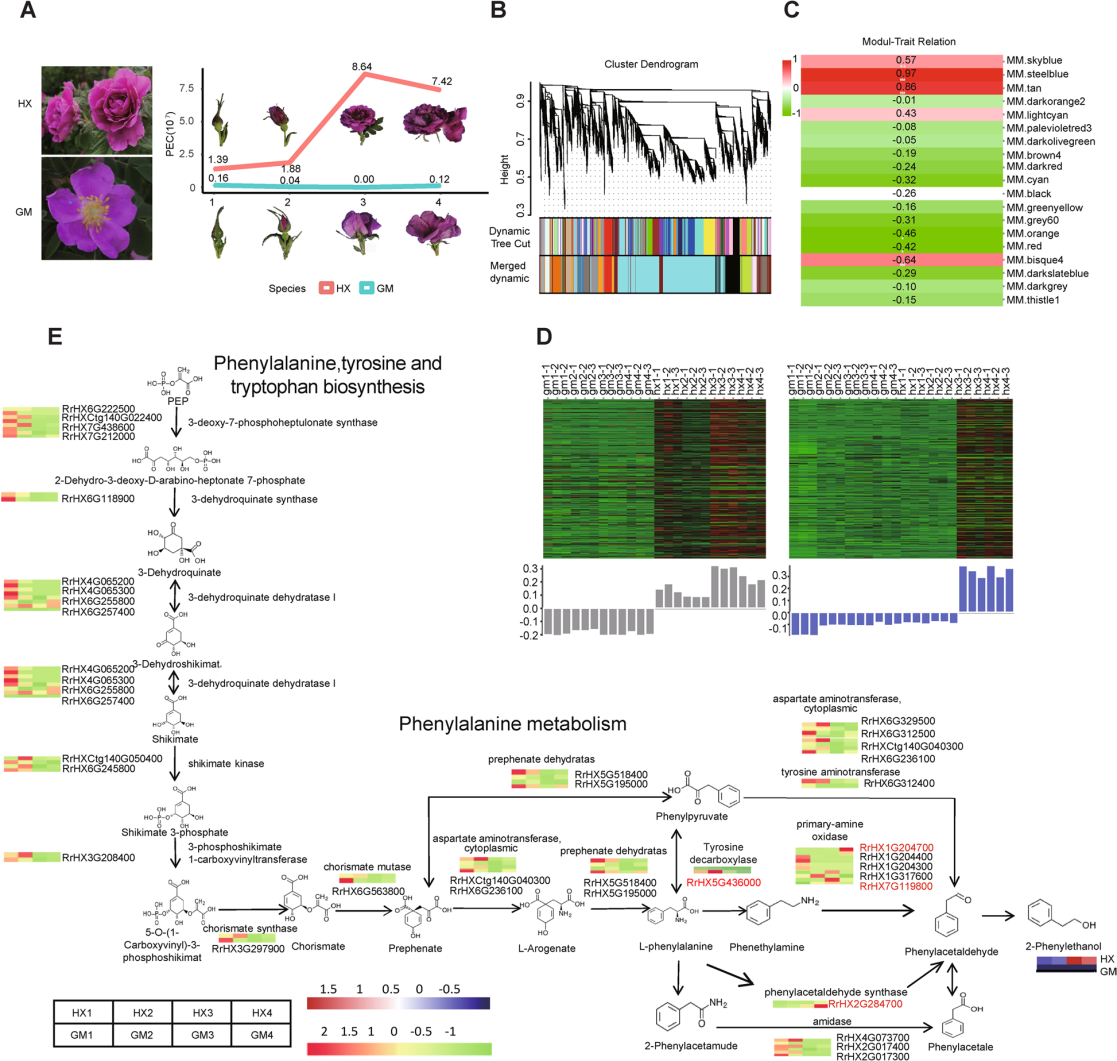
Gene expression and regulatory modules in fragrant and non-fragrant *R. rugosa* varieties. **A** Two *R. rugosa* varieties (HX and GM) with significantly different PE contents (PECs) at four flower development stages. **B** Module hierarchical clustering tree. Dynamic Tree Cut presents the module division based on the clustering results, whereas Merged dynamic presents the module division. **C** Module–PEC relationship diagram. The number in each grid represents the correlation between the module and the PEC, with the *P*-value provided in parentheses. **D** Tan and steelblue modules, which comprised genes that were more highly expressed in the HX petals than in the GM petals. Heat maps present the expression profiles of the co-expressed genes (sample names on top) in the modules (labeled on top). Red and green represent up-regulated and down-regulated expression levels, respectively. Bar graphs (below the heat maps) present the consensus expression patterns of the co-expressed genes in each module. **E** Expression profile and transcriptional regulatory network associated with the modules with the opposite expression patterns in HX and GM at each flower development stage

Both HX and GM were selected for the WGCNA to characterize genes in the aroma synthesis pathway. Notably, the 2-phenylethanol (2-PE) content was significantly higher in HX than in GM (Table S20). To investigate the HX and GM gene regulatory networks during flower development stages (bud stage, initial opening period, bloom period, and fading period; a total of 24 samples) (Fig. 4A), we identified co-expressed gene sets via a WGCNA, with the 2-PE content selected as the phenotype. Twenty-one modules (9–6,839 genes) were identified in HX and GM (Fig. 4B-C, Table S21). Interestingly, 10 co-expression modules in HX and 10 co-expression modules in GM were highly correlated (|e|≥ 0.30) with flower development stages (Fig. 4C, Table S22). The genes in the tan and steelblue modules were more highly expressed in the HX petals than in the GM petals (Fig. 4D).

The petal transcriptional changes during flower development stages were compared between HX and GM to clarify the relationships between the 2-PE synthesis-related genes. The production of 2-PE is directly related to the phenylalanine, tyrosine, and tryptophan biosynthetic pathway as well as the phenylalanine metabolic pathway. The expression levels of 34 genes encoding 16 enzymes changed in HX and GM at different flower development periods (Fig. 4E).

### *Rose* population structure and pre-breeding improvement of the flower scent

**Fig. 5:**
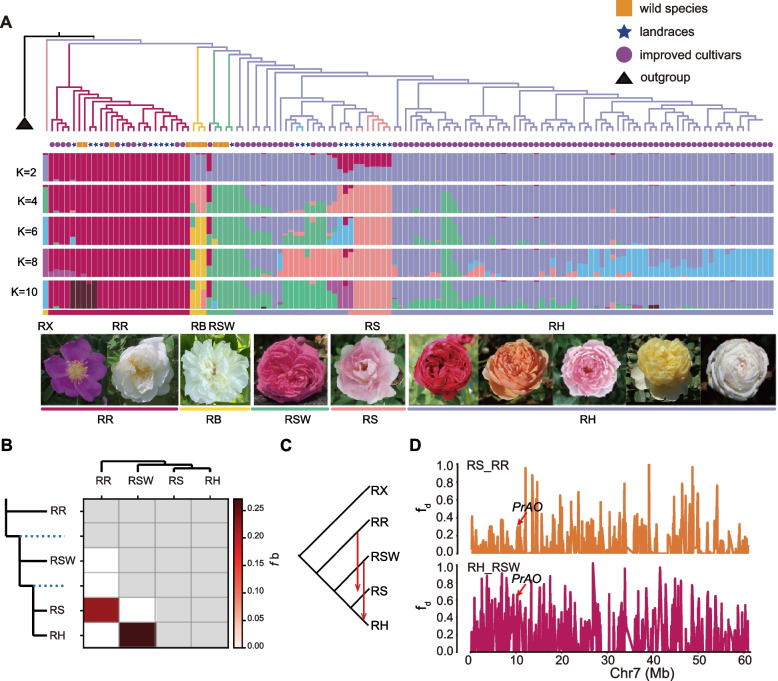
Population structure and genetic divergence among cultivated and wild *Rosa* species. **A** Group structure of 133 rose accessions, including 28 *R. rugosa* cultivars, 86 scented *R. hybrida* accessions, and 19 closely related fragrant *Rosa* species. The neighbor-joining tree was constructed and the model-based clustering analysis (K = 2, 4, 6, 8, and 10) was performed using 1,195,460 SNPs. **B** Results of *f*-branch for *Rosa* populations. The tree is displayed in an ‘expanded’ form along the y axis, so that each branch, including internal branches, points to a corresponding row in the matrix with inferred ƒ-branch statistics. **C** The phylogenetic topology used for inferring introgression between two group. **D** Introgression from donor populations to acceptor across chromosome 7 (acceptor_donor). The introgressed genes are labeled at corresponding positions on the chromosome

A total of 133 *Rosa* accessions were selected for genome resequencing with average sequencing depth of 13 ×, including 28 *R. rugosa* accessions, 19 fragrant *Rosa* species, and 86 scented *R. hybrida* cultivars (Table S24). We identified a total of 3,825,047 high-quality SNPs (Table S23). The neighbor-joining phylogenetic tree constructed using SNPs for the 133 *Rosa* accessions and five peach accessions (i.e., outgroup), the result separates the samples into two groups, group I (Sect. *Cinnamomeae* DC.) including most of the *R. rugosa* accessions and group II including all of the scented *R. hybrida* cultivars and fragrant *R.* species (Fig. 5A). The population structure was constructed under the model-based clustering analysis (*K* = 2, 3, 4, 5, and 6) (Fig. 5A). Group II was further divided into three subclades, consistent with their geographical distribution patterns. Group II-1 (Sect. *Banksianae* Lindl. and Sect. *Laevigatae* Thory) mainly contained accessions of southwestern wild species (RSWs and RBs) from China, group II-2 (Sect. *Chineses* DC.) mainly comprised scented *R. hybrida* cultivars (RHs), and group II-3 (Sect. *Rosa*) mainly included western varieties (RSs), primarily from Europe (e.g., *R.* × *centifolia* and *R.* × *damascena*) (Table S24). The *R. rugosa* (RR) accessions, including wild species, landraces, and improved cultivars, formed a monophyletic lineage, indicating that all currently grown *R. rugosa* accessions originated from a single domestication event (Fig. 5A). In addition, admixed ancestry was detected for some Rosa accessions, suggestive of introgression or gene flow during breeding.

We analyzed gene introgression among populations using Dsuite (Malinsky et al. 2021) and identified elevated ƒ-branch statistics between RR and RS, as well as between RSW and RH, which together form the genetic foundation of modern cultivated roses (Zhang et al. 2024). These findings suggest the presence of substantial gene flow signals between these two population pairs, indicating the potential for historical or ongoing genetic exchanges (Fig. 5B). We validated gene introgression between these populations using the D-statistic, and the significance of the D-stat and Z-scores confirmed the gene flow among these groups (Table S25). The results of gene flow were also presented through topological diagrams (Fig. 5C). We visualized the gene flow relationships among populations using phylogenetic topology. Further analysis of introgressed genes revealed that the *PrAO* gene, which is involved in the phenylalanine metabolism pathway, is located on Chr7 in RS-RR and RH-RSW introgression events (Fig. 5D, Table S26). Additionally, several other genes associated with the phenylalanine metabolism pathway, including *Bm1_28435*, *ELI5*, *At4g34880*, *TDC1*, *TDC2 and ASP1* were found to be introgressed between RS and RR populations on Chr1-Chr6 (Fig. S11). Given the pivotal role of the phenylalanine metabolism pathway in scent production, these findings suggest that RR contributed to the evolution of floral scent in the RS population.

### Gene variations related to 2-PE synthesis in the population and in other genomes

**Fig. 6:**
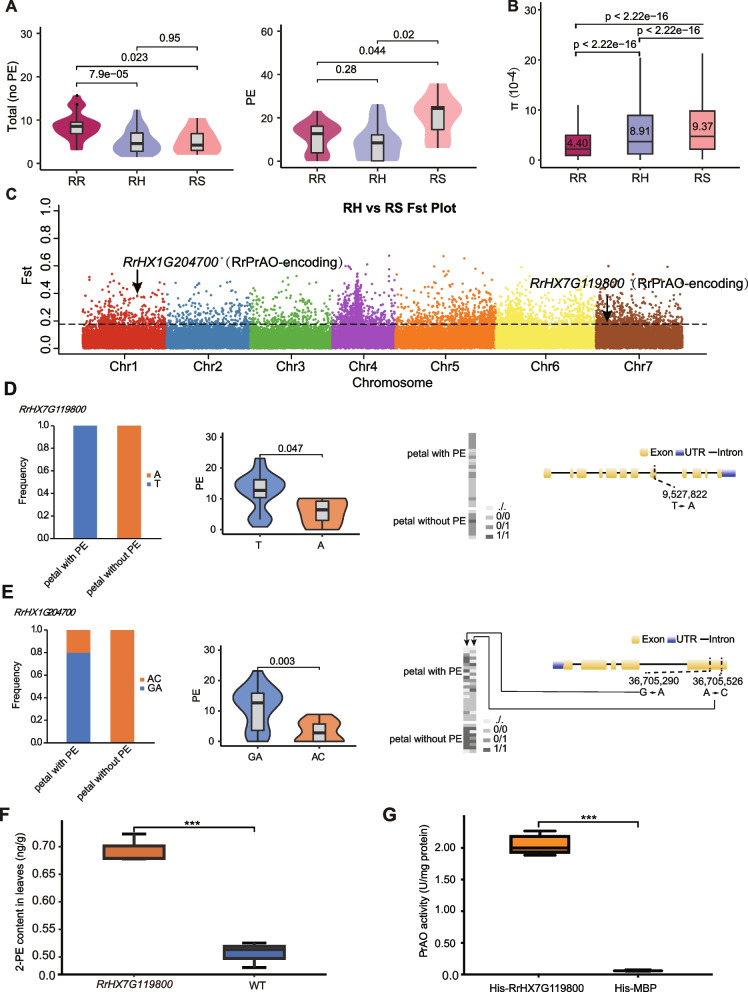
Variations in fragrance-related gene *RrHX7G119800* in the population and other genomes. **A** Aroma substance contents (not including PE) and PE contents in RR, RH, and RS. **B** Genetic diversity (θπ) for RR and RH. **C** *Fst* manhattan plot of RH vs RS. **D** *RrHX7G119800* gene structure and non-synonymous mutation sites*.* From left to right: haplotype frequency changes for *RrHX7G119800* in different groups, PE content for haplotypes T and A, genotypic distribution due to one non-synonymous SNP variation (‘/’ represents deletion, 0/0 represents genotype TT, 0/1 represents genotype TA, and 1/1 represents genotype AA). E *RrHX1G204700* gene structure and non-synonymous mutation sites*.* From left to right: haplotype frequency changes for *RrHX1G204700* in different groups, PE content for haplotypes GA and AC, genotypic distribution due to two non-synonymous SNP variations, and *RrHX1G204700* with different allelic expression patterns during four flowering periods

Focus on flower scent characteristics, sixteen major components of rose scent were detected by targeted metabolite analysis method, and 2-phenylethanol (2-PE), citronellol, nerol, linalool, rose ether etc., had great contribution to rose scent. The Principal Component Analysis (PCA) of SNP showed obvious divergence among RR, RS and RH groups, but the PCA of components (Fig. S12) showed slightly integration among three groups. These results suggest that the genetic differentiation of the three groups may be influenced by multiple traits. Compared with other accessions, RR petals contained more aroma substances, especially citronellol and rose ether, but their 2-phenylethanol content was less than that of RS petals (Fig. 6A, Table S23). Therefore, RR flowers may be enhanced by modulating the 2-PE content.

*R. rugosa* is one of the original parents of the modern cultivated rose species (i.e., RH) (Bendahmane et al. 2013). The π value (i.e., overall nucleotide diversity) of the 133 accessions was 7.83 × 10^−4^. The RS has the highest π value (9.37 × 10^−4^), the RH has higher π value (8.91 × 10^−4^), and RR showed lowest (4.40 × 10^−4^), indicative of no domestication bottleneck (Fig. 6B). To identify genomic regions under selection, we performed genome-wide *Fst* analysis using a sliding window of 10 kb with 5 kb steps. For each of the three pairwise comparisons (RH vs RR, RH vs RS, and RR vs RS), we selected the top 5% of weighted *Fst* values as thresholds (ranging from 0.1839 to 0.1840), identifying 1,599–1,613 putative selective sweeps that spanned 3,155–3,349 genes (Table S27). These candidate genes were significantly enriched in pathways such as phenylpropanoid biosynthesis, terpene synthase activity, and terpenoid biosynthetic process (Fig. S13). Notably, phenylpropanoid pathway genes were enriched in the RH vs RS comparison, suggesting artificial selection for floral scent traits. Among the candidate genes within selective sweeps, we identified two key primary amine oxidase (PrAO) genes, *RrHX1G204700.1* and *RrHX7G119800.1*, which may play roles in 2-PE biosynthesis (Fig. 6C).

In addition to genomic differentiation, we also quantified the content of 2-PE in rose petals, dividing the samples into two distinct groups based on their 2-PE concentrations. Genetic variation was then analyzed to identify loci associated with 2-PE content. Among the 34 genes encoding 16 enzymes involved in the 2-PE biosynthetic pathway, *RrHX1G204700* and *RrHX7G119800*, both encoding PrAO, were found to be significantly correlated with 2-PE levels (Fig. 6C). Furthermore, several non-synonymous (SNPs) in *RrHX7G119800* and *RrHX1G204700* gave rise to two major haplotypes. Specifically, non-synonymous SNPs in *RrHX7G119800* resulted in two distinct haplotypes, designated 'T' and 'A' (Fig. 6D). The frequency of ‘T’ increased in samples with increased PE contents (Fig. 6D). The accessions with ‘T’ had a significantly higher PE content (12.3514 μg/g, *P* < 0.05) than those with ‘A’ (5.7462 μg/g) (Table S28). Thus, ‘T’ was the favorable haplotype (Table S29). Similarly, two non-synonymous SNPs were identified in *RrHX1G204700*, with the 'GA' haplotype being more frequent in samples with higher PE content (Fig. 6E). Accessions carrying the 'GA' haplotype showed significantly higher PE levels (11.4039 μg/g, *P* < 0.01) compared to those with the 'AC' haplotype (3.2556 μg/g) (Supplementary Table S30). Thus, the 'GA' haplotype was also identified as a favorable variant associated with increased 2-PE content.

Moreover, we blast the gene *RrHX7G119800* in Rchi, PRr, and BT annotation genes. The structure of this gene is conserved in most species (Fig. S14), and they most have twelve cds, only the gene in *R. chinensis* has one shorter CDS than other species. In terms of gene sequences, there are a lot of variation between species (Fig. S15). The strawberry’s 2g01360.1 may caused by assembly problem. In addition to strawberries, we also found two copies of the this gene in both *P. pyrifolia* and *M. domestica*. A comparative analysis of *RrTDC*-encoding gene *RrHX5G43600* was also performed (Fig. S16).

In 2-PE synthesis pathway, the expression level of the *RrPrAO*-encoding gene *RrHX7G119800* was up-regulated in the third flowering stages of HX that consistent to 2-PE content trends (Fig. 4E). To analyze the function of the gene, transgenic tobacco plants overexpressing *RrHX7G119800* were generated (Fig. S17A), and ten independent transgenic lines were obtained. RT-PCR analysis of three transgenic lines showed that the expression level of *RrHX7G119800* was significantly higher than in wild-type plants (Fig. S17B). Subsequently, the 2-phenylethanol (2-PE) content in the leaves of wild-type and three transgenic lines was measured using GC–MS. The results revealed that the *RrHX7G119800* transgenic lines exhibited improved 2-PE content relative to WT plants (Fig. 6F), indicating that overexpression of the gene can promote the synthesis of 2-PE, which is consistent with the results of gene expression analysis. Additionally, we observed that these transgenic tobacco flowers emitted a subtle fragrance, further validating the role of *RrHX7G119800* in the synthesis of volatile compounds. To evaluate the enzymatic function of RrHX7G119800, we performed an in vitro PrAO activity assay using recombinant His-tagged proteins. The enzyme activity of His-RrHX7G119800 was significantly higher than that of the His-MBP control, indicating that RrHX7G119800 possesses primary amine oxidase activity. Specifically, His-RrHX7G119800 catalyzed the production of H_2_O_2_ at an average rate exceeding 2.0 U/mg protein, while the control group exhibited negligible activity (< 0.05 U/mg protein). This research outcome provides strong evidence for the discovery of a primary amine oxidase encoding gene in *R. rugosa*, and it offers novel genetic resources for enhancing aroma in other plant species.

## Discussion

The cultivation of rose species in China started in the Western Han Dynasty (202 BC–9 AD). The crosses within *R. rugosa* individuals and clonal propagation for approximately 2,000 years resulted in the heterozygosity and accumulation of somatic mutations in the genome, allowing us to separate the two haplotypes and identify allelic imbalances. Compared with earlier rose genomes such as PRr and *R. chinensis*, the HX assembly exhibits higher completeness and more extensive gene family expansion, particularly in pathways related to glutathione metabolism, photosynthesis, and aromatic amino acid biosynthesis. Of particular interest are two PAT-family genes, *RrHX6G236100.1* and *RrHXCtg140G040300.1*, associated with phenylalanine biosynthesis and potentially contributing to enhanced 2-PE production in HX. These findings provide improved genomic context for understanding floral scent biosynthesis in *R. rugosa*. Phylogenetic analysis further revealed that HX and PRr diverged approximately 4.5 million years ago, while *R. chinensis* and BT split earlier (~ 6.3 Mya), indicating distinct evolutionary trajectories among major rose species.

Allelic expression imbalance analysis classified ASE genes into consistent and inconsistent types. The asymmetrical expression of selected alleles was studied on the basis of the HX and GM transcriptome data at four flowering stages. In contrast to GM (with a low PE content), there were considerably more inconsistent ASE genes than consistent ASE genes in HX (10,596 *vs* 1,247), suggesting that the overdominance effect is a major reason for the high heterozygosity of the *R. rugosa* genome. The large abundance of inconsistent ASE genes in rose plants is likely caused by the accumulation of somatic mutations over a long period of clonal propagation, similar to what occurred in hybrid rice (Shao et al. 2019), but not in tea plants (Zhang et al. 2021). In addition, the haplotype frequency changes of genes related to 2-PE synthesis in different groups and the differences in these genes between the HX genome and other rose genomes were explored. The results suggested that ASE frequency changes affected gene expression, which resulted in phenotypic alterations.

Rose species have been modified by extensive reticulate evolution and interspecific hybridization, introgression, and polyploidization (Fougere-Danezan, 2015). Approximately 8–20 *Rosa* species contributed to the genetic make-up of modern rose cultivars, including *R. rugosa*, *R. chinensis*, *R. multiflora*, and *R. gigantea* as well as European rose species *R. moschata*, *R. gallica*, *R. canina*, and *R. phoenicia* (De Vries & Dubois, 1995; Reynders-Aloisi & Bollereau, 1996). Recent studies on the origin of modern roses suggested that the origin of modern roses results from hybridization and genetic contributions from multiple wild rose species, particularly *R. chinensis* 'Old Blush' and *R. odorata*, which together form the genetic foundation of modern cultivated roses (Zhang et al. 2024). In this study, we further validated significant gene introgression events between southwestern wild species (RSW) and scented *R. hybrida* cultivars (RH), which are consistent with previous reports (Zhang et al. 2024). Additionally, we identified gene flow events between *R. rugosa* (RR) and western varieties (RS) (Fig. 5B-C).This discovery highlights both historical and ongoing genetic exchanges that have likely shaped key phenotypic traits, particularly those related to floral scent (Ding et al. 2022).

The major scent components of *R. rugosa* and European rose flowers are 2-PE and several monoterpenes (e.g., rose oxide, geraniol, and nerol). In contrast, the *R. chinensis* floral fragrance is primarily due to lipid-derived alcohols and esters (e.g., hexenol and hexenyl acetate) and aromatic compounds (e.g., 3,5-dimethoxytoluene and 1,3,5-trimethoxybenzene) (Shi & Zhang 2022). Previous research confirmed 2-PE belongs to the second largest class of floral compounds (i.e., phenylpropanes) that produce a “honey-like” odor (Shao et al. 2019). Several natural 2-PE biosynthesis-related pathways have been identified, including the Ehrlich pathway, phenylethylamine pathway, and phenylpyruvate pathway. In previous studies, the genome of *R. hybrida* has been identified as a mosaic composed of multiple parental genomes (Bendahmane et al. 2013), with its fragrance traits predominantly attributed to *R. chinensis* ‘Old Blush’ (Raymond et al. 2018). In our experiments, we conducted an fd statistic analysis on populations undergoing gene introgression. We found that *PrAO*, located on chromosome 7, plays a crucial role in the phenylalanine metabolism pathway by catalyzing the conversion of phenylacetaldehyde into 2-PE, a key compound essential for floral aroma (Fig. 5D). Additionally, other introgressed scent-related genes, including *Bm1_28435*, *ELI5*, *At4g34880*, *TDC1*, *TDC2*, and *ASP1*, were identified on Chr1–Chr6 in RS and RR populations. These findings suggest that gene flow from RR to RS contributed to the enhancement of floral scent traits, thereby increasing the ecological and ornamental value of western varieties.

The biosynthetic pathway leading to 2-PE production was previously studied in detail and the PAAS (Farhi et al. 2010; Hirata et al. 2012; Kaminaga et al. 2006; Miwa et al. 2007) and PAR (Chen et al. 2011) was identified as key enzymes of the pathway. Moreover, an alternative pathway, which is seasonally induced in summer, has been identified in roses for the production of 2-PE (Hirata et al. 2016). This pathway uses Aromatic Amino Acid Aminotransferase (Miwa et al. 2007) and Phenylpyruvate Decarboxylase (Hirata et al. 2016) to produce 2-phenylacetaldehyde, whereas under the catalysis of PAAS enzyme, L-Phe is directly converted to phenylacetaldehyde (Farhi et al. 2010; Hirata et al. 2012; Miwa et al. 2007). However, we found the gene *RrHX7G119800* in *R. rugosa* encodes the RrPrAO protein, which is positively correlated with 2-PE content (high expression in bloom period petals of HX). Meanwhile tyrosine decarboxylase (RrTDC)-encoding gene *RrHX5G436000* was present in the *R. rugosa* genome. This should be an alternative pathway of 2-PE synthesis in *R. rugosa* that differs from other published *R. spp*. Phenylalanine is converted to phenylethylamine by tyrosine decarboxylase (RrTDC, encoding gene *RrHX5G436000*), and then to phenacetaldehyde by primary amine oxidase (RrPrAO, encoding gene *RrHX7G119800*). Previous studies have shown that primary amine oxidase (EC 1.4.3.21), also known as cu-amine oxidase (CAO, EC 1.4.3.6) (Klema et al., 2013; Moschou et al. 2012), belongs to amine oxidase (AOs), which was widely present in plants, animals, bacteria, yeast and fungi and played a major role in the metabolism of biological amines (Yu et al. 2020). It was also known as 2-phenylethylamine oxidase, and participates in the phenylethylamine pathway (Heli et al. 2015). Achmon et al. (2014) introduced rose-derived PAAS into *Escherichia coli*, achieving production of 0.39 g/L 2-PE in two steps, which shortened the fermentation time compared with ferment in yeast. The study and discovery of the natural pathway of primary amine oxidase and its coding genes in *R. rugosa* can also provide a good source of effective enzymes for constructing genetically engineered strains. Future studies should aim to identify while also clarifying the regulation of the core genes affecting phenylethanol synthesis in rose species, which may be relevant for optimizing the 2-PE content in rose flowers and expected to be used in the industrial production of 2-PE. The integration of transcriptomic and metabolomic data provides a powerful tool for uncovering complex scent biosynthesis networks, as shown in other terpenoid-rich aromatic plants (Liu et al., 2024; Zhou et al., 2024).

In conclusion, this study enhances our understanding of the molecular biology of rose fragrance, particularly by emphasizing the role of gene introgression and allelic imbalances in shaping floral scent traits. By elucidating the unique 2-PE synthesis pathway in *R. rugosa*, this research opens new avenues for optimizing fragrance composition in roses. Furthermore, the discovery of the natural 2-PE biosynthesis pathway in *R. rugosa* not only offers valuable genetic resources but also provides potential enzyme sources for industrial 2-PE production. Future research should focus on further exploring the regulatory mechanisms of core genes involved in phenylethanol synthesis. This will offer crucial insights into optimizing 2-PE content in rose flowers and expanding its applications in industrial production.

## Materials and methods

### Plant materials

The rose (*Rosa*) accessions used in this study were grown and collected from May to July 2021. The primary genome sequencing and two haplomes were conducted on *R. rugosa* cv. Hanxiang (HX), characterized by its strong floral scent. All accessions, including those used for genome, transcriptome, and resequencing analyses, were cultivated in the *Rosa* germplasm resource nursery of the Beijing Academy of Agriculture and Forestry Sciences, located in Beilangzhong, Zhaoquanying, and Shunyi, Beijing, China.

For resequencing and metabolite profiling, we selected 133 *Rosa* accessions. These accessions represent a wide spectrum of genetic backgrounds and fragrance profiles. The cultivar names, species information, and classification details are fully listed in Table S24.

### Genome sequencing

For the de novo assembly, samples were collected from a single plant. For the CCS analysis, genomic DNA was extracted from in vitro seedlings using the DNeasy Plant Mini Kit (Qiagen, Shanghai, China). A 15-kb library was constructed and sequenced using the Pacific Bioscience Sequel II platform (Annoroad Gene Technology). A total of 16.61 Gb CCS reads with an N50 value of 18.68 kb were generated using the ccs software (v.3.0.0) (https://github.com/pacificbiosciences/unanimity/; –min-passes 3 –min-length 10,000 –max-length 1,000,000 –min-rq 0.99). For the Hi-C analysis, leaf tissues were fixed in 1% formaldehyde before constructing libraries. After verifying the quality of the libraries was acceptable, they were sequenced using the Illumina HiSeq NovaSeq platform as previously described (Lieberman-Aiden et al. 2009). Briefly, the DpnII restriction enzyme was used for digesting chromatin. Libraries constructed using the Illumina TruSeq DNA Sample Prep Kit were sequenced using the Illumina NovaSeq Xten system to produce 2× 150-bp reads (25.93 Gb). The RNA-seq analysis was completed using root, stem, leaf, petal, stamen, pistil, sepal, and petiole samples collected for each accession. Total RNA was isolated using the RNAprep Pure Plant Kit (TIANGEN). The RNA-seq libraries were constructed according to a published method (Liu et al. 2017) and then sequenced on the NovaSeq platform.

### Genome size estimation

The genome size was estimated on the basis of a k-mer frequency analysis. The k-mer distribution depends on genome characteristics and follows Poisson’s distribution. Before the genome was assembled, the 17-mer distribution of WGS reads was determined using Jellyfish (v.2.2.6) (Marçais & Kingsford 2011). Additionally, GCE (Liu et al., 2013) was used to estimate genome characteristics.

Flow cytometry protocol, Firstly, take a sample of one-month-old tomato seedlings (reference genome size 0.88 G) and ginkgo as the samples. Then, place the samples in a pre-cooled MGb dissociation solution, quickly dice the tissue and let it sit on ice, then filter through a 400-mesh filter to obtain a cell nucleus suspension. Add a suitable amount of pre-cooled propidium iodide (PI) (stock concentration 1 mg/mL) and an appropriate volume of RNAase solution (stock concentration 1 mg/mL) to the cell nucleus suspension, stain on ice for 0.5–1 h in the dark. Mix the stained cell nucleus suspension with an appropriate ratio of internal reference sample and detect it using flow cytometry. Use BD FACScalibur flow cytometer, excite at 488 nm blue light, detect the fluorescence intensity of propidium iodide, and collect 10,000 particles for each detection. Finally, use Modifit 3.0 analysis software to plot the data.

Genome size calculation: Firstly, detect the fluorescence peak values of the test sample and the internal reference plant PI-DNA complex using flow cytometry, determine the ratio of DNA content between the two plants. Then, according to the known C value of the internal reference plant, use the calculation formula: test sample DNA content = internal reference DNA content x (test sample fluorescence intensity/internal reference fluorescence intensity) to calculate the DNA content of the test plant, i.e. its genome size.

### Chromosome preparation and karyotype analysis

Young leaf buds of *R. rugosa* cv. Hanxiang (HX) were collected for cytological karyotype analysis. Actively dividing root tip–like tissues from the buds (0.5–1 cm in length) were pretreated in a mixed solution of 0.001 M 8-hydroxyquinoline and 0.02% colchicine (1:1, v/v) at 4 °C in the dark for 4 h. The samples were then washed with distilled water and treated in 0.075 M KCl solution at 4 °C for 30 min for hypotonic treatment. After washing, tissues were fixed in Carnoy’s solution (methanol:acetic acid = 3:1, v/v) at 4 °C for 4–24 h. Fixed samples were then hydrolyzed with 1 M HCl at room temperature for 45 min and enzymatically digested in a mixture of 6% cellulase and 4% pectinase (2:1, v/v) at 37 °C for 5–6 h. After digestion, samples were washed with distilled water and further treated in a 37 °C water bath for 30 min. Chromosome spreads were prepared by squashing the digested tissue on clean glass slides. The chromosomes were stained with carbol fuchsin solution for 2 h. Coverslips were gently tapped with an eraser tip to spread the chromosomes uniformly. Well-spread metaphase cells were observed under a Nikon 80i microscope, and digital images were captured using a CCD camera. Chromosome measurements and karyotyping were conducted using Zeiss Karyotype software. A total of 14 chromosomes (2n = 2x = 14) were observed and classified into 10 metacentric and 4 submetacentric chromosomes, forming the karyotype formula 10 m + 4sm.

### Genome assembly

The contig genome was assembled using HiFi reads and Hi-C reads in hifiasm software Hi-C models: ‘-l 2 -k 51 -w 51 –h1 –h2’. We used p_ctg.fa as the monoploid contig result and hic.hap1.p_ctg.fa and hic.hap2.p_ctg.fa as the hap 1 and hap 2 contig results. The contigs were anchored to chromosomes using the Hi-C reads, which were aligned to the contigs using HICUP (v.0.7.3) (Wingett et al. 2015). The contigs were clustered using the ALLHiC (Zhang et al. 2018) algorithm (ALLHiC_partition -e GATC -k 7). Finally, the assembled genomes were manually corrected using Juicebox (v.1.11.08) (Burton et al. 2013). BUSCO (Simão et al. 2015) and LAI (Ou et al. 2018) were used to evaluate completeness.

### Genome annotation

Repeating elements were predicted via de novo and homology-based approaches. RepBase database was screened for sequence matches (http://www.girinst.org/repbase), after which repeating elements were predicted using RepeatProteinMask (http://www.repeatmasker.org/). LTR_FINDER(Xu & Wang 2007), RepeatScout (http://www.repeatmasker.org/), and Repeat-Modeler (http://www.repeatmasker.org/RepeatModeler.html) were used to construct a de novo library, which was then annotated using RepeatMasker (http://repeatmasker.org/).

Combining ab initio predictions, protein-based homology searches, and RNA sequencing was used to elucidate gene structures. Protein sequences from *R. chinensis*, *R. multiflora*, *R. rugosa*, *F. vesca*, *M. domestica*, and *P. persica* were aligned to the corresponding genome using WUblast (She et al. 2009), with an E-value cutoff of 1e-5. The resulting hits were conjoined using the Solar software(Wingett et al. 2015). GeneWise (Zhang et al. 2018) was used to predict the precise gene structures. Gene structures created by GeneWise were denoted as homology-based prediction gene set. Gene models created using PASA (Haas et al. 2003) were denoted as the PASA-T-set and used as the training data for the following five ab initio gene prediction programs that were used to predict coding regions in the repeat-masked genome: Augustus (v.2.5.5) (Stanke et al., 2006), Genscan (v.1.0) (Burge & Karlin 1997), GeneID (Guigo 1998), GlimmerHMM (v.3.0.1) (Majoros et al. 2004), and SNAP (Korf 2004). The RNA-seq data (Table S28) were mapped to the assembly using TopHat (v.2.0.8) (Kim et al. 2013) and then Cufflinks (v.2.1.1) (Ghosh & Chan 2016) was used to assemble the transcripts into gene models (Cufflinks-set). In addition, the gene models predicted from the Trinity-assembled transcripts using PASA were designated as the PASA-T-set (PASA Trinity set). Gene model evidence from the Homo-set, PASA-Iso-set, Cufflinks-set, PASA-T-set, and ab initio data was combined using EVidenceModeler (Haas et al. 2008) into a non-redundant set of gene annotations. The weight of each type of evidence was as follows: PASA-T-set > Homo-set > Cufflinks-set > Augustus > GeneID = SNAP = GlimmerHMM = Genscan.

The predicted protein sequences were functionally annotated using the following five databases: NR, InterPro, GO, KEGG, and Swiss-Prot. The InterPro and GO databases were searched using the following parameters of InterProScan (Hunter et al., 2009): “-f TSV -dp -gotermes -iprlookup”. The NR, Swiss-Prot, and KEGG databases were searched using BLAST, with an E-value cut-off of 1 × 10^−5^. The results from these database analyses were concatenated.

### SV identification

Hap1 was aligned to hap 2 using the following parameters of mummer: –maxgap = 500 –mincluster = 500. The raw alignments were filtered using the following parameters of delta-filter: -i 95 -l 1000 −1. After using the “show-coords”, the filtered results were converted to an easy-to-read file. This file was used to detect structural variations using the default parameters of the SyRI (Goel et al. 2019) pipeline: –no-chrmatch –nosnp.

### Identification of alleles

Interhaplotype syntenic blocks were identified using the default parameters of MCScan. The paired genes corresponding to the same blocks were considered to be alleles.

### Dominant allele expression

Petals in different flowering periods were collected from mature plants for an RNA-seq analysis. The generated RNA-seq reads were trimmed using fastp (Chen et al. 2018) and then mapped to the combined genomes (hap1 and hap2) using HISAT2. The FPKM values were calculated on the basis of unique mapped reads using the featureCounts software and a homemade R script. To analyze dominant allele expression, we extracted 21,033 genes with known alleles. The allele pairs were compared to analyze differential expression.

### SNP and small InDel calling

We collected Illumina resequencing data for 133 newly sequenced *Rosa* accessions (average depth of 13 ×). The raw resequencing data were screened for high-quality reads using the default settings of fastp (v.0.20.1) (Chen et al. 2018). To detect SNPs, Illumina short reads were aligned to the rose genome using BWA-MEM; PCR duplicates were removed using Picard (v.1.118) (http://broadinstitute.github.io/picard/). The SNPs and InDels were identified using HaplotypeCaller from the Genome Analysis Toolkit (v.4.1.5.0) (McKenna et al. 2010) and then filtered as previously described (Guo et al. 2019). The SNPs with a read depth < 5 and non-biallelic SNPs were eliminated.

### Preparation of transcriptome sequencing samples

Petals were collected from *R. rugosa* cv. Hanxiang (HX) and *R*. *rugosa* Thunb. f. *rosea* Rehd. (GM) plants at four flowering stages for the subsequent RNA extraction. Analyses were completed using the following replicates:HX 1–1, HX 1–2, HX 1–3, HX 2–1, HX 2–2, HX 2–3, HX 3–1, HX 3–2, HX 3–3, HX 4–1, HX 4–2, HX 4–3, GM 1–1, GM 1–2, GM 1–3, GM 2–1, GM 2–2, GM 2–3, GM 3–1, GM 3–2, GM 3–3, GM 4–1, GM 4–2, and GM 4–3.

### Dsuite, D-stat and fd statistic

To detect introgression events between two groups, we used Dsuite software (Malinsky et al. 2021).First, genomic data were extracted from Variant Call Format (VCF) files, with an outgroup specified to define ancestral alleles. Next, D statistics and *f*_4_-ratios were calculated using Dtrios to detect and quantify gene flow signals among population trios. In sliding window analysis, Dinvestigate was employed to evaluate whether gene flow was confined to specific genomic regions and to compute related statistics (e.g., *f*_*d*_ and *f*_*d*_*M*). Finally, Fbranch was used to attribute gene flow to specific branches of the phylogenetic tree, producing matrices to display the strength of gene flow across branches.

To verify the introgression events detected by Dsuite, we applied the ABBA–BABAtest (Dstatistic) by detecting differences in allele sharing between two lineages (P1 and P2) with a third lineage (P3) (Durand et al. 2011). RX was used as the outgroup. D-stat greater than 0 means that there is gene flow between P1 and P3; If the D-stat is less than 0, there is a gene between P2 and P3. Subsequently, the fd statistic (Martin et al. 2014) was computed to identify introgressed genomic fragments based on the tree form (((P1, P2), P3), O), consistent with the above Dstatistic. The fd statistic was computed in 100-kb windows and 50-kb step with the python script ABBABABAwindows.py (https://github.com/simonhmartin/genomics_general).

### RNA extraction, cDNA library construction, and Illumina sequencing

Total RNA was extracted from the leaf samples and used to prepare sequencing libraries for the RNA-seq analysis. Specifically, total RNA was isolated using the TRIzol reagent according to the manufacturer’s protocol (TaKaRa, Dalian, China). Eukaryotic mRNA in the extracted total RNA was enriched using oligo-(dT) beads. The cDNA was then purified using the QIAquick PCR Purification Kit. A poly-A sequence was added to the blunt-ended cDNA, which was then ligated to the Illumina sequencing linker. The ligation product of the desired size was selected by agarose gel electrophoresis, amplified by PCR, and sequenced using the Illumina HiSeq™ 4000 system at Gene Denovo Biotechnology Co. (Guangzhou, China).

### Read mapping and differential gene expression analysis

The retained high-quality clean reads (Q-value ≤ 10) were mapped to rRNA to identify residual RNA reads. After eliminating the rRNA reads, the remaining high-quality clean reads were mapped to the reference transcriptome using the default parameters of Bowtie2 (Li et al. 2009). The mapping rate was calculated using the following equation: mapping rate = (number of unique mapped reads + number of multiple mapped reads)/total number of reads.

Gene expression levels were calculated and normalized as RPKM (reads per kilobase per million reads) values (Mortazavi et al. 2008) using the following formula: RPKM = 106 × C/(NL/103), where C is the number of reads uniquely aligned to a specific gene, N is the total number of reads uniquely aligned to all genes, and L is the number of bases in the specific gene. Calculating gene expression according to the RPKM value eliminated the influence of gene length and sequencing data abundance. The edge R package (http://www.r-project.org/) was used to identify differentially expressed genes (DEGs) across samples or groups. An expression level fold-change ≥ 2 and a false discovery rate < 0.05 were used as the thresholds for identifying significant DEGs, which were then subjected to GO and KEGG pathway enrichment analyses.

### Gene expression analysis

Gene expression patterns were analyzed to cluster the genes similarly expressed in multiple samples (i.e., at least three at a specific time-point). To examine the DEG expression patterns, the expression data for each sample were normalized to 0, log_2_ (v1/v0), and log_2_ (v2/v0) and then clustered using the Short Time-series Expression Miner software(Ernst & Bar-Joseph 2006). The parameters were set as follows: maximum unit change in model profiles between time-points was 1; maximum output profiles was 20 (similar profiles were merged); and the minimum DEG expression level fold-change was ≥ 2.0. A *P*-value ≤ 0.05 was used as the threshold for identifying significant cluster profiles. The DEGs in the profiles were subjected to GO and KEGG pathway enrichment analyses. An adjusted *P*-value based on a false discovery rate ≤ 0.05 (Benjamini & Hochberg 1995) was used to identify the significantly enriched GO terms and KEGG pathways.

### Gas chromatography–mass spectrometry and statistical analyses of targeted metabolomics and volatile metabolomics data

Fresh petal samples were collected from 27 *R. rugosa* cultivars, 43 scented *R. hybrida* cultivars, and 7 fragrant *Rosa* species used in the targeted metabolomics analysis are fully listed in Table S24. The samples were ground to a powder in liquid nitrogen and transferred to a 20 mL headspace vial (Agilent, Palo Alto, CA, USA) containing NaCl saturated solution to inhibit enzymatic reactions. The vials were sealed using crimp-top caps with TFE–silicone headspace septa (Agilent) prior to the HS-SPME. The VOCs were desorbed from the fiber coating in the injection port of the GC apparatus (Model 8890; Agilent) at 250 °C for 5 min in the splitless mode. The VOCs were identified and quantified using the Model 8890 GC system (Agilent) and the 5977B MS system (Agilent) equipped with a 30 m × 0.25 mm × 0.25 µm DB-5MS (5% phenyl-polymethyl siloxane) capillary column. Mass spectra were scanned in the range *m*/*z* 30–350 amu at 1 s intervals. Volatile compounds were identified by comparing the mass spectra with the data system library (MWGC) and linear retention indices.

We extracted citronellol, phenethyl alcohol, farnesol, nerol, and rose oxide from petals by HS-SPME for the targeted metabolomics analysis using the GC–MS system (Cheng et al. 2022; Feng et al. 2022). The analysis was completed as previously described (Raymond et al. 2018; Yuan et al., 2018; Yun et al. 2016; Zhou et al. 2015). The metabolites were sorted according to their content and concentration. On the basis of the International Standard for Oil of Rose (ISO 9843: 2003), we compared the most important metabolites with published data for the following 16 target metabolites: eight terpenes (α-farnesene, nerolidol, nerol, farnesol, rose ether, citronellal, citronellol, and linalool), four esters (neryl acetate, citronellyl acetate, phenethyl acetate, and geranyl acetate), two phenols (eugenol and methyleugenol), one alcohol (phenethyl alcohol), and one heterocyclic compound (*trans*-linalool oxide). We prepared standard curves for the 16 target substances and quantified their contents.

The HX and GM *R. rugosa* accessions with differing fragrances were selected for the volatile metabolomics analysis at four flower development stages. We used the Mass Hunter software (Agilent) to extract and analyze the original data for the volatile metabolites identified by GC–MS and then analyzed the data qualitatively, quantitatively, and statistically. We prepared the quality control (QC Mix) sample by combining the extracts of all samples to assess the repeatability of the results.

### Co-expression network analysis for constructing modules

Co-expression networks were constructed using the WGCNA (v.1.47) package in R (Langfelder & Horvath 2008). After filtering genes (i.e., removing more than 40% of the genes according to their RPKM values), the gene expression values were imported into WGCNA to construct co-expression modules using the automatic network construction function blockwiseModules. The default settings were used, with the following exceptions: power was 10, TOM Type was unsigned, mergeCutHeight was 0.7, and minModuleSize was 50. Genes were clustered into 21 correlated modules. For each module, a summary profile (module eigengene) was calculated via a PCA. To evaluate the association between the modules and stage-specific expression in each cultivar, we determined the correlation between each module eigengene and the 2-PE content in petals as previously described (Downs et al. 2013).

### Genotyping of the population comprising samples with differing 2-PE contents

The ASE genes related to phenylethanol synthesis were screened. The SNPs resulting in non-synonymous mutations were extracted, and the SNPs with a heterozygosity greater than 0.2 and a maf less than 0.05 were filtered. The filtered SNPs were used for the genotyping of the samples with varying 2-PE contents. The genes with different major haplotype frequencies in the two populations were selected. The corresponding phenotypes of all samples with different haplotypes were examined using the *t*-test, after which the genes associated with significantly different phenotypes were retained.

### Tobacco transgenic experimentation protocol

Initially, plump tobacco seeds are selected and subjected to sterilization treatment before being inoculated onto a culture medium for aseptic seedling cultivation. Concurrently, Agrobacterium is propagated through streak culture method on LB solid medium containing 100 mg/L Spectinomycin or Kanamycin, 50 mg/L Gentamicin, and 50 mg/L Rifampicin. After incubation at 28 °C for 2 days, a single colony is picked and inoculated into LB liquid medium containing antibiotics to prepare an Agrobacterium suspension with an OD600 = 0.4–1.2. Once the aseptic seedlings reach 1–2 months of age, tender leaves are harvested, cut into small pieces, and prepared as explants. These explants are then immersed in the Agrobacterium suspension for 8–10 min and placed on 1/2MS medium, followed by dark cultivation at 25 °C for 2–4 days. After co-cultivation, the explants are transferred to 1/2 MS medium containing 100 mg/L Kanamycin to induce bud differentiation. Resistant plants are obtained through rooting selection. Finally, genomic DNA is extracted using the SDS method for PCR detection to verify the transformation results.

### In vitro PrAO activity assay

PrAO activity was assessed by a colorimetric assay based on H_2_O_2_ production (Angelini et al. 2018). Purified His-tagged RrPrAO protein (1 μg) was incubated with 150 mM putrescine (Put) in 100 mM sodium phosphate buffer (pH 7.0) at 37 °C for 40 min in a 100 μL reaction volume. The reaction was terminated with 10 μL of 20% (w/v) trichloroacetic acid (TCA) and centrifuged at 12,000 × g for 10 min at 4 °C. A 20 μL aliquot of the supernatant was mixed with 160 μL phosphate buffer (100 mM, pH 7.0), 10 μL 4-aminoantipyrine (100 mM), 5 μL DCHBS (1 mM), and 5 μL horseradish peroxidase (1 U/mL). After 15 min of incubation in the dark at room temperature, absorbance was measured at 515 nm. H_2_O_2_ concentration was calculated using a standard curve (0–7 μM). One unit (U) of activity was defined as the formation of 1 μmol H_2_O_2_ per minute. Results were expressed as U/mg protein. Data are presented as mean ± SD (*n* = 3), and statistical analysis was performed by one-way ANOVA (*p* < 0.05).

## Supplementary Information

Supplementary Material 1: Figure S1. K-mer analysis and flow cytometry result. Figure S2. Inter-chromosomal Hi-C contact map of each chromosome. Figure S3. Karyotype of HX based on leaf bud mitotic cells. Figure S4. Three assemblies’s BUSCO reasults. Figure S5.LAI distribution of each chromosome of the three genomes with a window length of 3M.Figure S6. The collinearity of the three genomes with *R. rugosa*. Figure S7. Coverage of three genomes by the Illumina reads.Figure S8. Comparison of gene structural features (gene length, CDS length, exon length) of monoploid, hap1 and hap2. Figure S9. Analysis of the synteny between the *R. chinensis* genome assembly, monoploid (HX) genome assembly, and PRr genome assembly. Figure S10. The ASE gene’s expression in petals of hap1 and hap2 in bud stage (hx 1), initial opening period (hx 2), and fading period (hx 4) of HX. Figure S11. Images of *R. rugosa* GM plant parts. Figure S12. Introgression from donor populations to acceptor across chromosome 1-6 (acceptor_donor). Figure S13. The Principal Component Analysis of RR, RH and RS. Figure S14. Selected genes’ enrichment results. Figure S15. Evolutionary tree of *RrHX7G119800* and gene structure. Figure S16. *RrHX7G119800* homologous gene sequence comparison. Figure S17. *RrHX5G43600* homologous gene sequence comparison. Figure S18. Overexpressing *RrHX7G119800* in transgenic tobacco plants.

Supplementary Material 2: Table S1. Statistics of genomic features obtained by Kmer=17 analysis. Table S2. Genome size estimates from flow cytometry. Table S3. Summary of genome assembly and annotation of *R. rugosa *HX. Table S4. The mounting rate of the genomes. Table S5. Evaluation of three genomes compared with PRr. Table S6. Hap1's general statistics of predicted protein-coding genes. Table S7. Hap2's general statistics of predicted protein-coding genes. Table S8. Monoploid genome's general statistics of predicted protein-coding genes. Table S9. Statistics of gene functional annotation. Table S10. Summary of the transposon element for three genomes. Table S11. Summary of the ncRNA for hap1. Table S12. Summary of the ncRNA for hap2. Table S13. Summary of the ncRNA for monoploid genome. Table S14. SV dection summary. Table S15. ASE summary. Table S16. Transcription factors prediction of ASE gene influenced by SV. Table S17. GO and KEGG enrich of allele-specific expression genes. Table S18. Enriched biological system pathways of specifically expressed alleles of HX (stage3). Table S19. ASE type of phenylalanine synthesis pathway related gene. Table S20. ANOVA (analysis of variance) of PE content between HX and GM. Table S21. Number of genes in co-expression modules. Table S22. Modular eigenvalues of module genes in each sample. Table S23. ANOVA annotation of snps. Table S24. 133 newly sequenced *Rosa* accessions of *R. rugosa* (RR), scented *R. hybrida* (RH), and fragrant *R. *species (RB, RSW, RSP, RS, and RX). Table S25. The result of Dstat. Table S26. FD introgression fragment' statistics. Table S27. Three group *F *st selected result statistics. Table S28. FPKM of *RrHX7G119800* in different period. Table S29. Variations statistics of *RrHX7G119800*. Table S30. Variations statistics of *RrHX1G204700*.

## Data Availability

Data will be available from the corresponding author upon reasonable request.

## Abbreviations

*BUSCO*: Benchmarking Universal Single-Copy Orthologs
*LAI*: LTR Assembly Index
L-phenylalanine: L-Phe
PAAS: Phenylacetaldehyde synthase
PAR: Phenylacetaldehyde reductase
PrAO: Primary amine oxidase
*VOCs*: Volatile organic compounds
*WGCNA*: Weighted Gene Co-expression Network Analysis

## References

[CR1] Achmon Y, Zelas BB, Fishman A. Cloning *Rosa* hybrid phenylacetaldehyde synthase for the production of 2-phenylethanol in a whole cell *Escherichia coli* system. Appl Microbiol Biotechnol. 2014;98(8):3603–11. 24081322 10.1007/s00253-013-5269-z

[CR2] Angelini R, Cona A, Tavladoraki P. Determination of Copper Amine Oxidase Activity in Plant Tissues. Methods Mol Bio. 2018;1694:129–39. 29080163 10.1007/978-1-4939-7398-9_13

[CR3] Bendahmane M, Dubois A, Raymond O, Bris ML. Genetics and genomics of flower initiation and development in roses. J Exp Bot. 2013;64(4):847–57. 23364936 10.1093/jxb/ers387 PMC3594942

[CR4] Benjamini Y, Hochberg Y. Controlling the false discovery rate: a practical and powerful approach to multiple testing. J R Stat Soc Series B Stat Methodol. 1995;57(1):289–300.

[CR5] Białecka-Florjańczyk E, Krzyczkowska J, Stolarzewicz I, Kapturowska A. Synthesis of 2-phenylethyl acetate in the presence of *Yarrowia lipolytica* KKP 379 biomass. J Mol Catal B Enzym. 2012;74(3–4):241–5. 10.1016/j.molcatb.2011.10.010.

[CR6] Burge C, Karlin S. Prediction of complete gene structures in human genomic DNA. J Mol Biol. 1997;268(1):78–94. 9149143 10.1006/jmbi.1997.0951

[CR7] Burton JN, Adey A, Patwardhan RP, Qiu R, Kitzman JO, Shendure J. Chromosome-scale scaffolding of de novo genome assemblies based on chromatin interactions. Nat Biotechnol. 2013;31(12):1119–25. 24185095 10.1038/nbt.2727 PMC4117202

[CR8] Çelik D, Bayraktar E, Mehmetoğlu Ü. Biotransformation of 2-phenylethanol to phenylacetaldehyde in a two-phase fed-batch system. Biochem Eng J. 2004;17(1):5–13.

[CR9] Chen XM, Kobayashi H, Sakai M, Hirata H, Asai T, Ohnishi T, et al. Functional characterization of rose phenylacetaldehyde reductase (PAR), an enzyme involved in the biosynthesis of the scent compound 2-phenylethanol. J Plant Physiol. 2011;168(2):88–95. 20650544 10.1016/j.jplph.2010.06.011

[CR10] Chen S, Zhou Y, Chen Y, Gu J. Fastp: an ultra-fast all-in-one FASTQ preprocessor. Bioinformatics. 2018;34(17):i884–90. 30423086 10.1093/bioinformatics/bty560 PMC6129281

[CR11] Chen F, Su L, Hu S, Xue JY, Liu H, Liu G, et al. A chromosome-level genome assembly of rugged rose (Rosa rugosa) provides insights into its evolution, ecology, and floral characteristics. Hortic Res. 2021;8:141. 34145222 10.1038/s41438-021-00594-z PMC8213826

[CR12] Cheng X, Feng Y, Chen D, Luo C, Yu X, Huang C. Evaluation of Rosa germplasm resources and analysis of floral fragrance components in R. rugosa. Front Plant Sci. 2022;13:1026763. 36311132 10.3389/fpls.2022.1026763 PMC9597504

[CR13] China MI. Compendium of materia medica. 2014.

[CR14] Commission CP. Pharmacopoeia of the People’s Republic of China: Volume I (2005). Beijing: People’s Medical Publishing House; 2005.

[CR15] Committee NP. Pharmacopoeia of the People’s Republic of China. 2015;Part 1:188–189.

[CR16] DeVries D, Dubois LA. Rose breeding: past, present, prospects. II International Rose Symposium. 1995:424.

[CR17] Ding YM, Cao Y, Zhang WP, Chen J, Liu J, Li P, et al. Population-genomic analyses reveal bottlenecks and asymmetric introgression from Persian into iron walnut during domestication. Genome Biol. 2022;23(1):1–18. 35787713 10.1186/s13059-022-02720-z PMC9254524

[CR18] Downs GS, Bi YM, Colasanti J, Wu W, Chen X, Zhu T, et al. A developmental transcriptional network for maize defines coexpression modules. Plant Physiol. 2013;161(4):1830–43. 23388120 10.1104/pp.112.213231 PMC3613459

[CR19] Durand EY, Patterson N, Reich D, Slatkin M. Testing for ancient admixture between closely related populations. Mol Biol Evol. 2011;28:2239–52. 21325092 10.1093/molbev/msr048 PMC3144383

[CR20] Ernst J, Bar-Joseph Z. STEM: a tool for the analysis of short time series gene expression data. BMC Bioinformatics. 2006;7(1):1–11. 16597342 10.1186/1471-2105-7-191 PMC1456994

[CR21] Farhi M, Lavie O, Masci T, Hendel-Rahmanim K, Weiss D, Abeliovich H, et al. Identification of rose phenylacetaldehyde synthase by functional complementation in yeast. Plant Mol Biol. 2010;72(3):235–45. 19882107 10.1007/s11103-009-9564-0

[CR22] Feng Y, Cheng X, Lu Y, Wang H, Chen D, Luo C, et al. Gas chromatography-mass spectrometry analysis of floral fragrance-related compounds in scented rose (*Rosa hybrida*) varieties and a subsequent evaluation on the basis of the analytical hierarchy process. Plant Physiol Biochem. 2022;185:368–77. 35753285 10.1016/j.plaphy.2022.06.007

[CR23] Fougere-Danezan LB. Phylogeny and biogeography of wild roses with specific attention to polyploids. Ann Bot. 2015;115(2):275. 10.1093/aob/mcu245 PMC4551085 25550144

[CR24] Ghosh S, Chan CK. Analysis of RNA-Seq data using TopHat and Cufflinks. Methods Mol Biol. 2016;1374:339–61. 10.1007/978-1-4939-3167-5_18. 26519415 10.1007/978-1-4939-3167-5_18

[CR25] Goel M, Sun H, Jiao WB, Schneeberger K. SyRI: finding genomic rearrangements and local sequence differences from whole-genome assemblies. Genome Biol. 2019;20(1):1–13. 31842948 10.1186/s13059-019-1911-0 PMC6913012

[CR26] Guigo R. Assembling genes from predicted exons in linear time with dynamic programming. J Comput Biol. 1998;5(4):681–702. 10072084 10.1089/cmb.1998.5.681

[CR27] Gunaseelan S, Balupillai A, Govindasamy K, Muthusamy G, Ramasamy K, Shanmugam M, et al. The preventive effect of linalool on acute and chronic UVB-mediated skin carcinogenesis in Swiss albino mice. Photochem Photobiol Sci. 2016;15(7):851–60. 27251985 10.1039/c6pp00075d

[CR28] Guo S, Zhao S, Sun H, Wang X, Wu S, Lin T, et al. Resequencing of 414 cultivated and wild watermelon accessions identifies selection for fruit quality traits. Nat Genet. 2019;51(11):1616–23. 31676863 10.1038/s41588-019-0518-4

[CR29] Gurdal Orhan IO, Subutay-Oztekin N, Ak F, Sener B. Contemporary anticholinesterase pharmaceuticals of natural origin and their synthetic analogues for the treatment of Alzheimer’s disease. Recent Pat CNS Drug Discov. 2009;4(1):43. 10.2174/157488909787002582 19149713

[CR30] Haas BJ, Delcher AL, Mount SM, Wortman JR, Smith RK Jr, Hannick LI, et al. Improving the Arabidopsis genome annotation using maximal transcript alignment assemblies. Nucleic Acids Res. 2003;31(19):5654–66. 14500829 10.1093/nar/gkg770 PMC206470

[CR31] Haas BJ, Salzberg SL, Zhu W, Pertea M, Allen JE, Orvis J, et al. Automated eukaryotic gene structure annotation using EVidenceModeler and the Program to Assemble Spliced Alignments. Genome Biol. 2008;9(1):1–22. 10.1186/gb-2008-9-1-r7 PMC2395244 18190707

[CR32] Hancianu M, Cioanca O, Mihasan M, Hritcu L. Neuroprotective effects of inhaled lavender oil on scopolamine-induced dementia via anti-oxidative activities in rats. Phytomedicine. 2013;20(5):446–52. 23351960 10.1016/j.phymed.2012.12.005

[CR33] Heli E, Teija H, Mikael M, Kati E, Yegutkin GG, Mikael S, et al. Primary amine oxidase of Escherichia coli is a metabolic enzyme that can use a human leukocyte molecule as a substrate. PLoS ONE. 2015;10(11):e0142367. 26556595 10.1371/journal.pone.0142367 PMC4640556

[CR34] Hibrand Saint-Oyant L, Ruttink T, Hamama L, Kirov I, Lakhwani D, Zhou NN, et al. A high-quality genome sequence of *Rosa chinensis* to elucidate ornamental traits. Nat Plants. 2018;4(7):473–84. 29892093 10.1038/s41477-018-0166-1 PMC6786968

[CR35] Hirata H, Ohnishi T, Ishida H, Tomida K, Sakai M, Hara M, et al. Functional characterization of aromatic amino acid aminotransferase involved in 2-phenylethanol biosynthesis in isolated rose petal protoplasts. J Plant Physiol. 2012;169(5):444–51. 22236980 10.1016/j.jplph.2011.12.005

[CR36] Hirata H, Ohnishi T, Tomida K, Ishida H, Kanda M, Sakai M, et al. Seasonal induction of alternative principal pathway for rose flower scent. Sci Rep. 2016;6:20234. 26831950 10.1038/srep20234 PMC4735289

[CR37] Hunter S, Apweiler R, Attwood TK, Bairoch A, Bateman A, Binns D, et al. InterPro: the integrative protein signature database. Nucleic Acids Res. 2009;37(suppl_1):D211–15. 10.1093/nar/gkn785 PMC2686546 18940856

[CR38] Kaminaga Y, Schnepp J, Peel G, Kish CM, Ben-Nissan G, Weiss D, et al. Plant phenylacetaldehyde synthase is a bifunctional homotetrameric enzyme that catalyzes phenylalanine decarboxylation and oxidation. J Biol Chem. 2006;281(33):23357–66. 16766535 10.1074/jbc.M602708200

[CR39] Kim D, Pertea G, Trapnell C, Pimentel H, Kelley R, Salzberg SL. TopHat2: accurate alignment of transcriptomes in the presence of insertions, deletions and gene fusions. Genome Biol. 2013;14(4):1–13. 10.1186/gb-2013-14-4-r36 PMC4053844 23618408

[CR40] Klema VJ, Solheid CJ, Klinman JP, Wilmot CM. Structural analysis of aliphatic vs. aromatic substrate specificity in a copper amine oxidase from Hansenula polymorpha. Biochemistry. 2013;52(13):2291. 10.1021/bi3016845 PMC3633420 23452079

[CR41] Korf I. Gene finding in novel genomes. BMC Bioinformatics. 2004;5(1):1–9. 15144565 10.1186/1471-2105-5-59 PMC421630

[CR42] Langfelder P, Horvath S. WGCNA: an R package for weighted correlation network analysis. BMC Bioinformatics. 2008;9(1):1–13. 19114008 10.1186/1471-2105-9-559 PMC2631488

[CR43] Li R, Yu C, Li Y, Lam TW, Yiu SM, Kristiansen K, et al. SOAP2: an improved ultrafast tool for short read alignment. Bioinformatics. 2009;25(15):1966–7. 19497933 10.1093/bioinformatics/btp336

[CR44] Lieberman-Aiden E, Van Berkum NL, Williams L, Imakaev M, Ragoczy T, Telling A, et al. Comprehensive mapping of long-range interactions reveals folding principles of the human genome. Science. 2009;326(5950):289–93. 19815776 10.1126/science.1181369 PMC2858594

[CR45] Linck VM, da Silva AL, Figueiro M, Caramao EB, Moreno PRH, Elisabetsky E. Effects of inhaled linalool in anxiety, social interaction and aggressive behavior in mice. Phytomedicine. 2010;17(8–9):679–83. 19962290 10.1016/j.phymed.2009.10.002

[CR46] Liu Z, Li H, Wen Z, Fan X, Li Y, Guan R, et al. Comparison of genetic diversity between Chinese and American soybean (*Glycine max* (L.)) accessions revealed by high-density SNPs. Front Plant Sci. 2017;8:2014. 29250088 10.3389/fpls.2017.02014 PMC5715234

[CR47] Liu M, Li Y, Chen H, He C, Sun L, Zhang X, et al. Integrated omics profiles for exploring the potential mechanism underlying aroma formation in the terpenoid-rich aromatic plant *Opisthopappus taihangensis* and the bioactivity of its leaf essential oil. Agriculture Communications. 2024;2(4):100061.

[CR48] Liu B, Shi Y, Yuan J, Hu X, Zhang H, Li N, et al. Estimation of genomic characteristics by analyzing k-mer frequency in de novo genome projects. arXi. 2013;1308.2012v2.

[CR49] Majoros WH, Pertea M, Salzberg SL. Tigrscan and GlimmerHMM: two open source ab initio eukaryotic gene-finders. Bioinformatics. 2004;20(16):2878–9. 15145805 10.1093/bioinformatics/bth315

[CR50] Malinsky M, Matschiner M, Svardal H. Dsuite: fast D-statistics and related admixture evidence from VCF files. Mol Ecol Resour. 2021;21(2):584–95. 33012121 10.1111/1755-0998.13265 PMC7116594

[CR51] Marçais G, Kingsford C. A fast, lock-free approach for efficient parallel counting of occurrences of k-mers. Bioinformatics. 2011;27(6):764–70. 21217122 10.1093/bioinformatics/btr011 PMC3051319

[CR52] Martin SH, Davey JW, Jiggins CD. Evaluating the use of ABBA-BABA statistics to locate introgressed loci. Mol Biol Evol. 2014;32:244–57. 25246699 10.1093/molbev/msu269 PMC4271521

[CR53] McKenna A, Hanna M, Banks E, Sivachenko A, Cibulskis K, Kernytsky A, et al. The genome analysis toolkit: a MapReduce framework for analyzing next-generation DNA sequencing data. Genome Res. 2010;20(9):1297–303. 20644199 10.1101/gr.107524.110 PMC2928508

[CR54] Miwa S, Sakai H, Hirata H, Sayama K, Sekiguchi H, Itano M. Production of 2-phenylethanol in roses as the dominant floral scent compound from L-phenylalanine by two key enzymes, a PLP-dependent decarboxylase and a phenylacetaldehyde reductase. Biosci Biotechnol Biochem. 2007;71(10):2408–19. 17928708 10.1271/bbb.70090

[CR55] Mortazavi A, Williams BA, McCue K, Schaeffer L, Wold B. Mapping and quantifying mammalian transcriptomes by RNA-seq. Nat Methods. 2008;5(7):621–8. 18516045 10.1038/nmeth.1226 PMC13303166

[CR56] Moschou PN, Cona A, Tavladoraki P, Angelini R, Roubelakis-Angelakis KA. The polyamines and their catabolic products are significant players in the turnover of nitrogenous molecules in plants. J Exp Bot. 2012;63(14):5003–15. 22936828 10.1093/jxb/ers202

[CR57] Orhan G, Orhan I, Subutay-Oztekin N, Ak F, Sener B. Contemporary anticholinesterase pharmaceuticals of natural origin and their synthetic analogues for the treatment of Alzheimer’s disease. Recent Pat CNS Drug Discov. 2009;4(1):43–51. 19149713 10.2174/157488909787002582

[CR58] Ou S, Chen J, Jiang N. Assessing genome assembly quality using the LTR assembly index (LAI). Nucleic Acids Res. 2018;46(21):e126. 30107434 10.1093/nar/gky730 PMC6265445

[CR59] Raymond O, Gouzy J, Just J, Badouin H, Verdenaud M, Lemainque A, et al. The rosa genome provides new insights into the domestication of modern roses. Nat Genet. 2018;50(6):772–7. 29713014 10.1038/s41588-018-0110-3 PMC5984618

[CR60] Reynders-Aloisi S, Bollereau P. Characterisation of genetic diversity in genus *Rosa* by randomly amplified polymorphic DNA. Acta Hortic. 1996;424:253–60.

[CR61] Roccia A, Hibrand-Saint Oyant L, Cavel E, Caissard JC, Machenaud J, Thouroude T, et al. Biosynthesis of 2-phenylethanol in rose petals is linked to the expression of one allele of RhPAAS. Plant Physiol. 2019;179(3):1064–79. 30622153 10.1104/pp.18.01468 PMC6393788

[CR62] Senol FS, Orhan IE, Kurkcuoglu M, Khan MTH, Altintas A, Sener B, et al. A mechanistic investigation on anticholinesterase and antioxidant effects of rose (*Rosa damascena* Mill.). Food Res Int. 2013;53(1):502–9.

[CR63] Shang J, Feng D, Liu H, Niu L, Li R, Li Y, et al. Evolution of the biosynthetic pathways of terpene scent compounds in roses. Curr Biol. 2024;34:3550-63.e3558. 39043188 10.1016/j.cub.2024.06.075

[CR64] Shao L, Xing F, Xu C, Zhang Q, Che J, Wang X, et al. Patterns of genome-wide allele-specific expression in hybrid rice and the implications on the genetic basis of heterosis. Proc Natl Acad Sci U S A. 2019;116(12):5653–8. 30833384 10.1073/pnas.1820513116 PMC6431163

[CR65] She R, Chu JS-C, Wang K, Pei J, Chen N. GenBlastA: enabling BLAST to identify homologous gene sequences. Genome Res. 2009;19(1):143–9. 18838612 10.1101/gr.082081.108 PMC2612959

[CR66] Shi S, Zhang Z. Genetic and biochemical aspects of floral scents in roses. Int J Mol Sci. 2022;23(14):8014. 35887360 10.3390/ijms23148014 PMC9321236

[CR67] Simão FA, Waterhouse RM, Ioannidis P, Kriventseva EV, Zdobnov EM. BUSCO: assessing genome assembly and annotation completeness with single-copy orthologs. Bioinformatics. 2015;31(19):3210–2. 26059717 10.1093/bioinformatics/btv351

[CR68] Stanke M, Keller O, Gunduz I, Hayes A, Waack S, Morgenstern B. AUGUSTUS: ab initio prediction of alternative transcripts. Nucleic Acids Res. 2006;34(suppl_2):W435–39. 10.1093/nar/gkl200 PMC1538822 16845043

[CR69] Wang Y, Xue Y, Bi Q, Qin D, Du Q, Jin P. Enhanced antibacterial activity of eugenol-entrapped casein nanoparticles amended with lysozyme against gram-positive pathogens. Food Chem. 2021;360:130036. 34004594 10.1016/j.foodchem.2021.130036

[CR70] Wingett S, Ewels P, Furlan-Magaril M, Nagano T, Schoenfelder S, Fraser P, et al. HiCUP: pipeline for mapping and processing Hi-C data. F1000Res. 2015;4:1310. 26835000 10.12688/f1000research.7334.1 PMC4706059

[CR71] Xu Z, Wang H. Ltr_finder: an efficient tool for the prediction of full-length LTR retrotransposons. Nucleic Acids Res. 2007;35(suppl_2):W265–8. 17485477 10.1093/nar/gkm286 PMC1933203

[CR72] Yu T, Yin Y, Ge Y, Cheng S, Zhang X, Feng Z, et al. Enzymatic production of 4-hydroxyphenylacetaldehyde by oxidation of the amino group of tyramine with a recombinant primary amine oxidase. Process Biochem. 2020;92:105–12.

[CR73] Yuan N, Qiong X, Zhuang J, Yude W, Lilan D, Dengfei L, et al. Determination of aromatic components of Rosa davurica Pall. by headspace solid phase microextraction combined with GC-MS. Med Plant. 2018;9(5):2152–24.

[CR74] Yun MM, Li BY, Zhou XM. Determination of aromatic components of flower in Rosa rugosa Thunb. by the static headspace and gas chromatography-mass spectrometry technology. Sci Technol Food Ind. 2016.

[CR75] Zhang J, Zhang X, Tang H, Zhang Q, Hua X, Ma X, et al. Allele-defined genome of the autopolyploid sugarcane Saccharum spontaneum L. Nat Genet. 2018;50(11):1565–73. 30297971 10.1038/s41588-018-0237-2

[CR76] Zhang X, Chen S, Shi L, Gong D, Zhang S, Zhao Q, et al. Haplotype-resolved genome assembly provides insights into evolutionary history of the tea plant *Camellia sinensis*. Nat Genet. 2021;53(8):1250–9. 34267370 10.1038/s41588-021-00895-y PMC8346365

[CR77] Zhang Z, Yang T, Liu Y, Wu S, Sun H, Wu J, et al. Haplotype-resolved genome assembly and resequencing provide insights into the origin and breeding of modern rose. Nat Plants. 2024;10:1659–71. 39394508 10.1038/s41477-024-01820-x

[CR78] Zhong MC, Jiang XD, Yang GQ, Cui WH, Suo ZQ, Wang WJ, et al. Rose without prickle: genomic insights linked to moisture adaptation. Natl Sci Rev. 2021;8:nwab092 10.1093/nsr/nwab092 PMC8694671 34987840

[CR79] Zhou L, Wu S, Chen Y, Huang R, Cheng B, Mao Q, et al. Multi-omics analyzes of *Rosa gigantea* illuminate tea scent biosynthesis and release mechanisms. Nat Commun. 2024;15:8469. 10.1038/s41467-024-52782-9 PMC11443146 39349447

