# Supplementary material for: Haplotype-resolved genome assembly provides insights into the unique floral scent of Rosa rugosa originated in China
Source: Mol Hortic. 2026 Apr 7;6:27. doi: 10.1186/s43897-025-00210-x (PMC13055005; doi:10.1186/s43897-025-00210-x)
Supplement: Supplementary file 1 — Supplementary Material 1: Figure S1. K-mer analysis and flow cytometry result. Figure S2. Inter-chromosomal Hi-C contact map of each chromosome. Figure S3. Karyotype of HX based on leaf bud mitotic cells. Figure S4. Three assemblies’s BUSCO reasults. Figure S5.LAI distribution of each chromosome of the three genomes with a window length of 3M.Figure S6. The collinearity of the three genomes with R. rugosa. Figure S7. Coverage of three genomes by the Illumina reads.Figure S8. Comparison of gene structural features (gene length, CDS length, exon length) of monoploid, hap1 and hap2. Figure S9. Analysis of the synteny between the R. chinensis genome assembly, monoploid (HX) genome assembly, and PRr genome assembly. Figure S10. The ASE gene’s expression in petals of hap1 and hap2 in bud stage (hx 1), initial opening period (hx 2), and fading period (hx 4) of HX. Figure S11. Images of R. rugosa GM plant parts. Figure S12. Introgression from donor populations to acceptor across chromosome 1-6 (acceptor_donor). Figure S13. The Principal Component Analysis of RR, RH and RS. Figure S14. Selected genes’ enrichment results. Figure S15. Evolutionary tree of RrHX7G119800 and gene structure. Figure S16. RrHX7G119800 homologous gene sequence comparison. Figure S17. RrHX5G43600 homologous gene sequence comparison. Figure S18. Overexpressing RrHX7G119800 in transgenic tobacco plants. [file 43897_2025_210_MOESM1_ESM.docx]

**Supplement Figures**


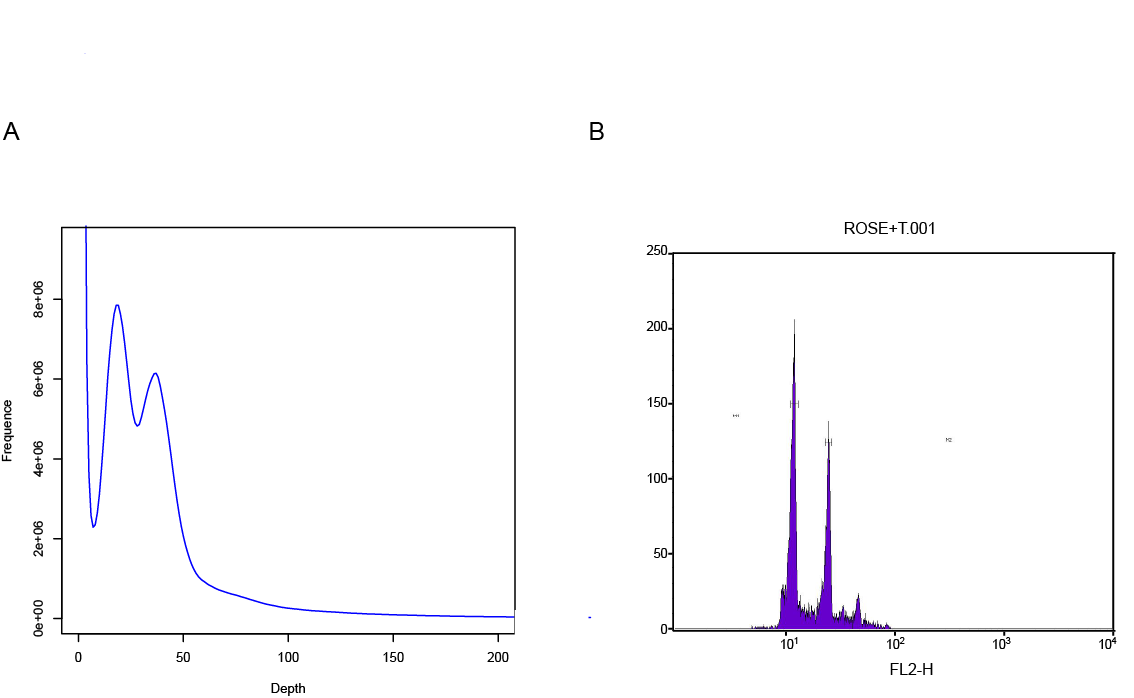


**Figure S1. K-mer analysis and flow cytometry result.**


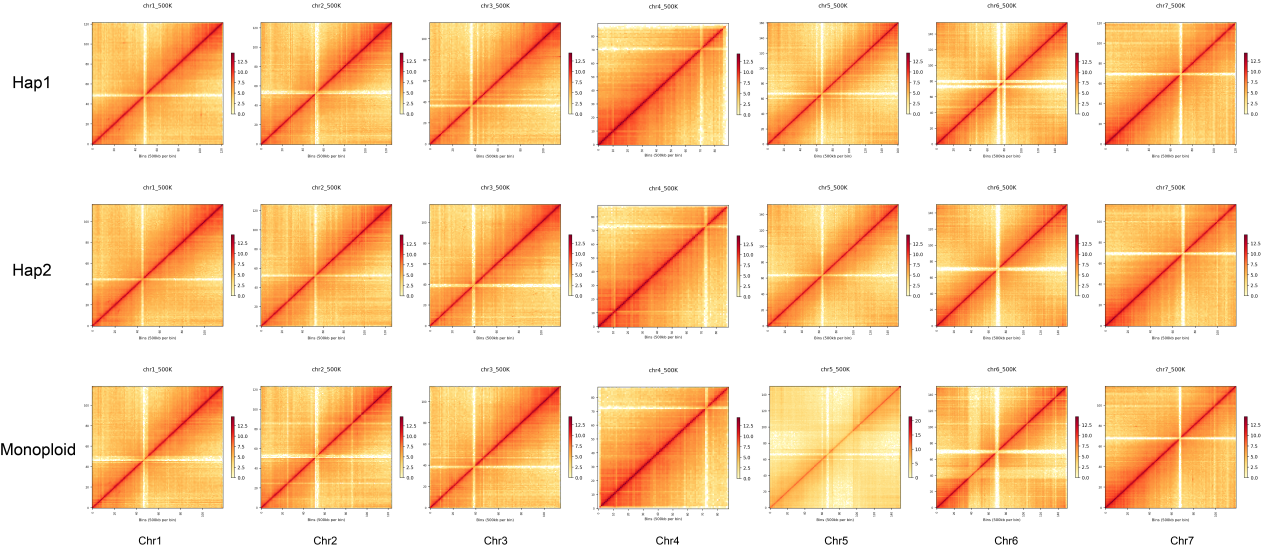


**Figure S2. Inter-chromosomal Hi-C contact map of each chromosome.**

The intensity of each pixel represents the count of Hi-C links between 500kb windows on chromosomes on a logarithmic scale. Darker red pixels indicate a higher contact probabilities.


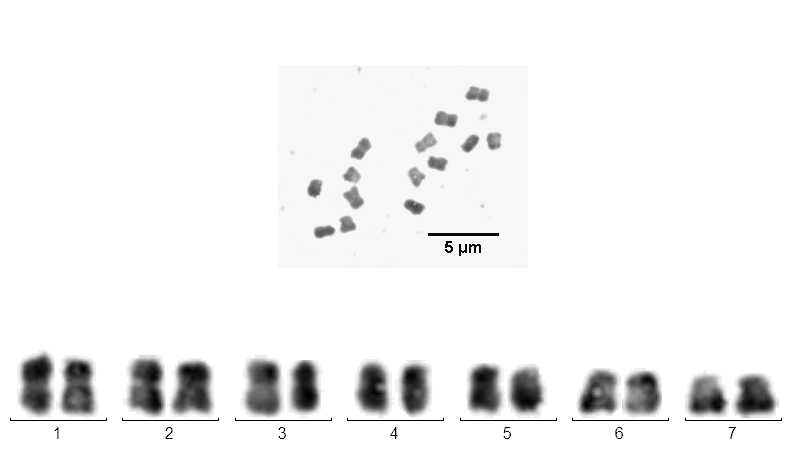


**Figure S3. Karyotype of HX based on leaf bud mitotic cells.**

Cytological karyotype of HX derived from leaf bud mitotic cells. The analysis revealed that HX is a diploid species with 2n = 2x = 14 chromosomes. Chromosomes were classified into 10 metacentric (m) and 4 submetacentric (sm) types, based on the karyotypic formula 10m + 4sm. Chromosome spreads were prepared using enzymatic digestion followed by carbol fuchsin staining, and observed under a Nikon 80i microscope. Karyotype image analysis was performed using Zeiss Karyotype software.


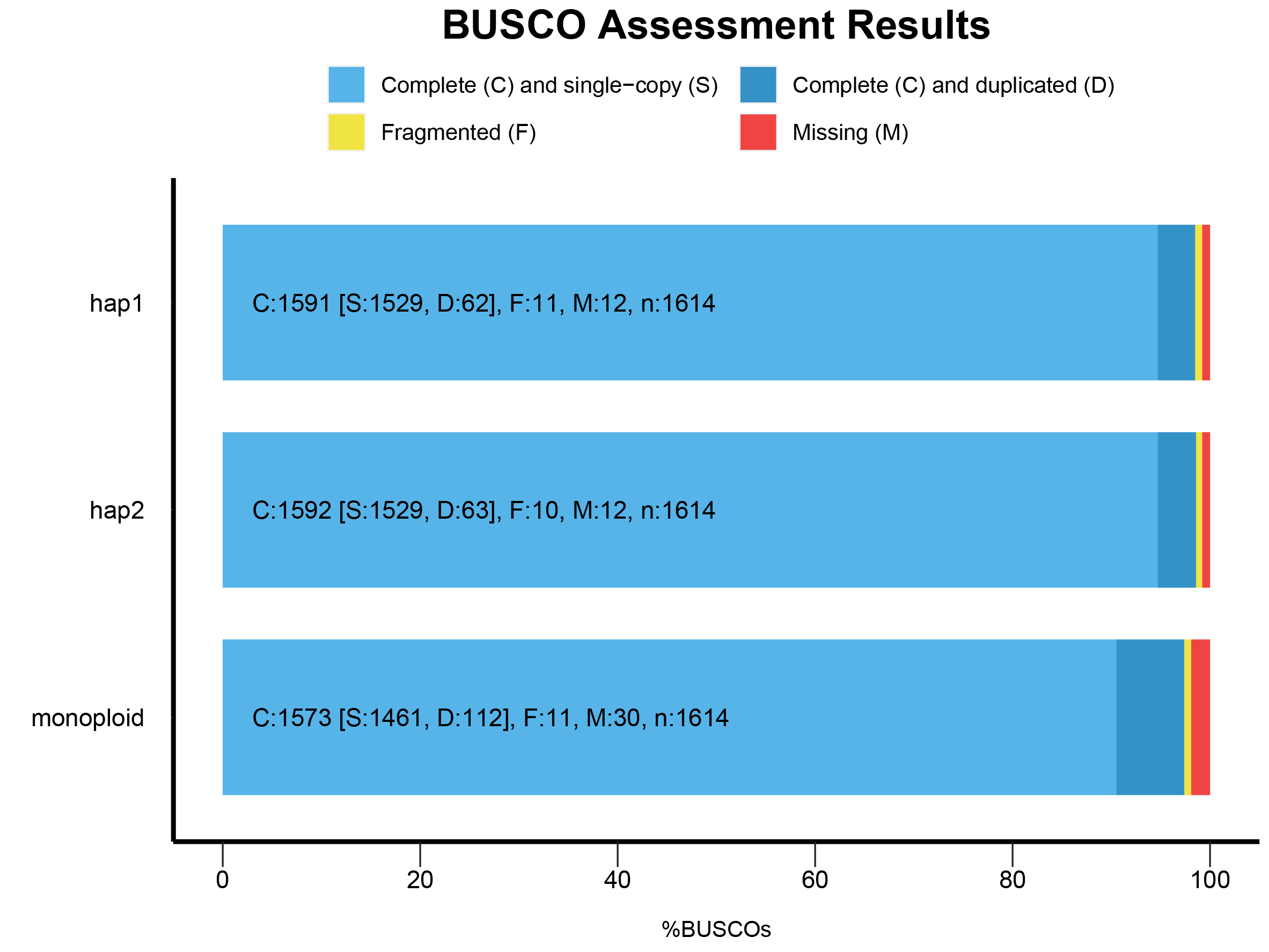


**Figure S4. Three assemblies’s BUSCO reasults.**


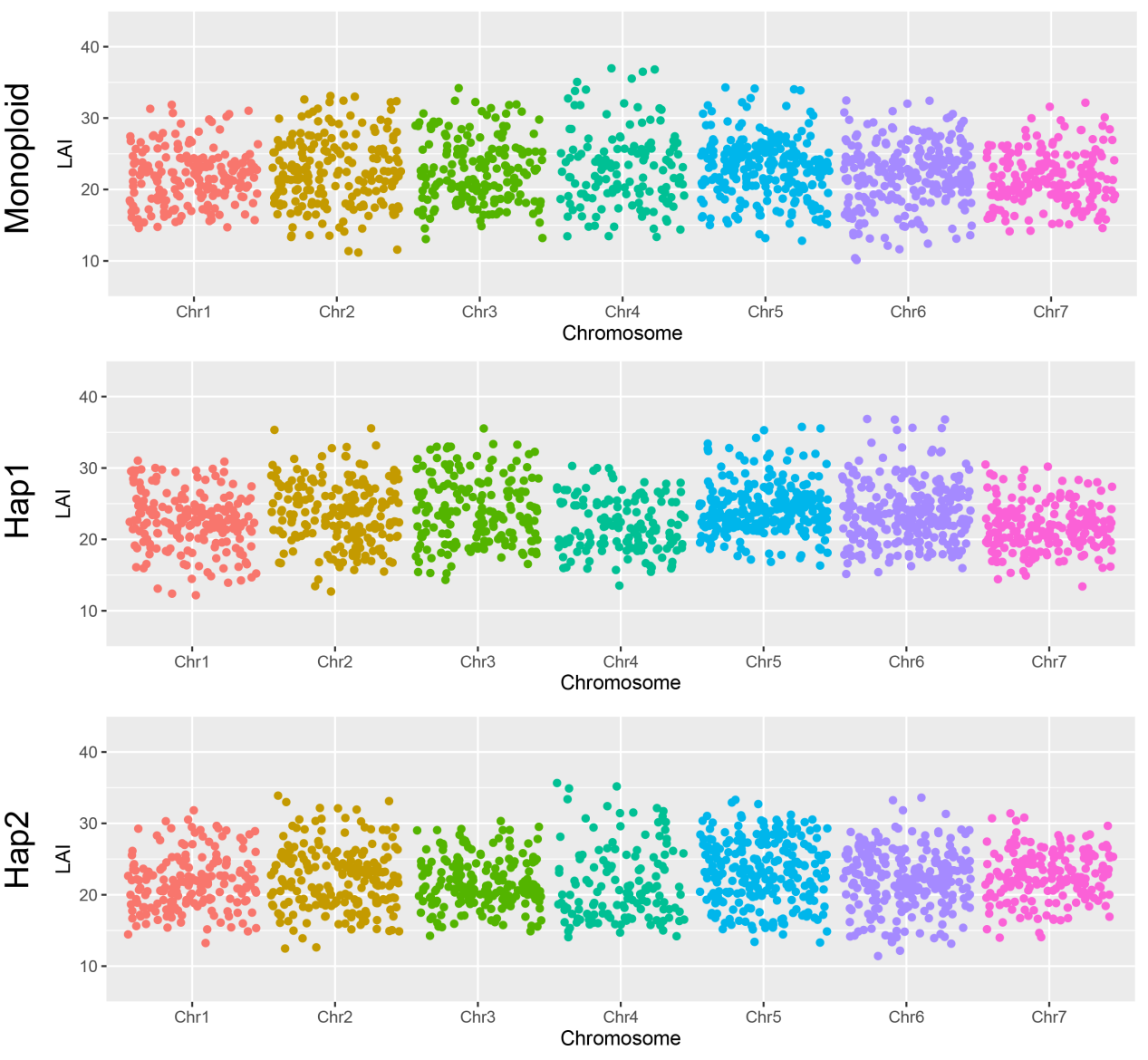


**Figure S5. LAI distribution of each chromosome of the three genomes with a window length of 3M.**


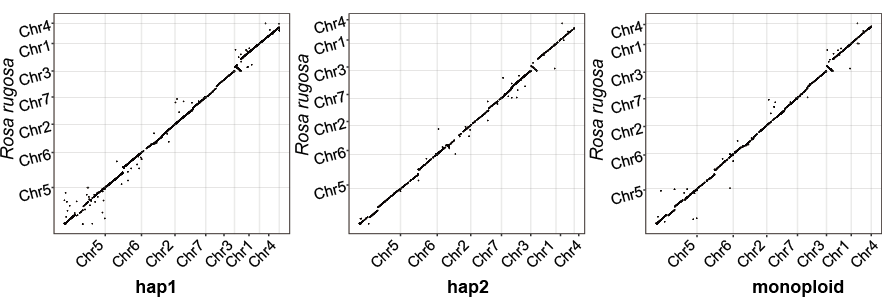


**Figure S6. The collinearity of the three genomes with *R. rugosa.***


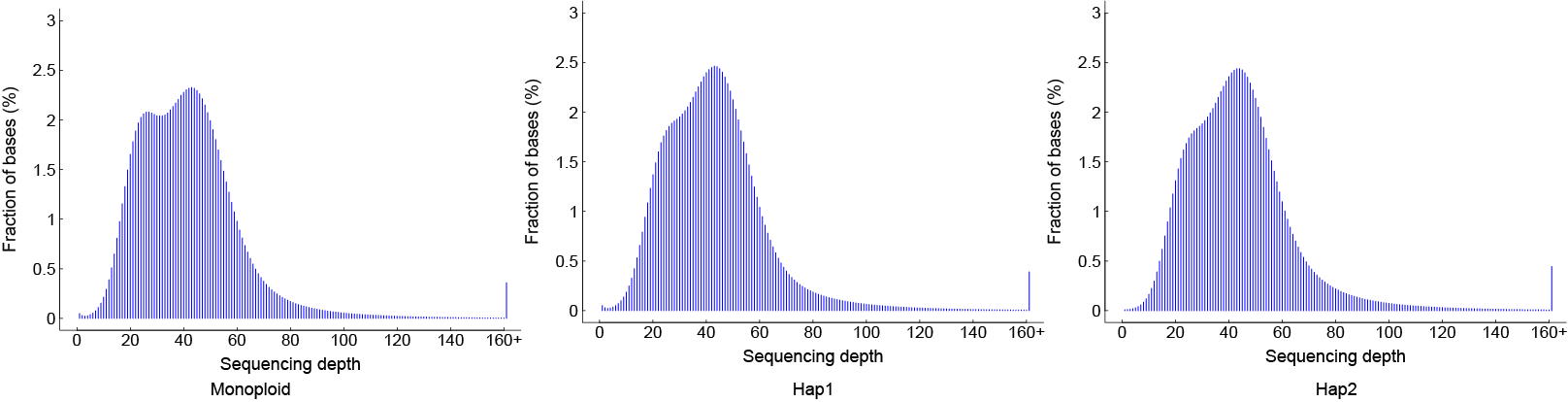


**Figure S****7. Coverage of three genomes by the Illumina reads.**


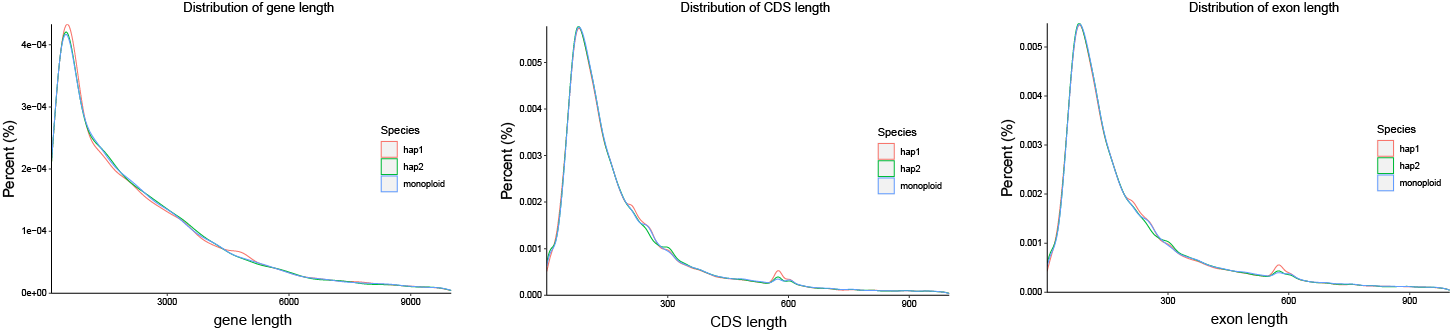


**Figure S8. Comparison of gene structural features (gene length,CDS length,exon length) of monoploid , hap1 and hap2.**


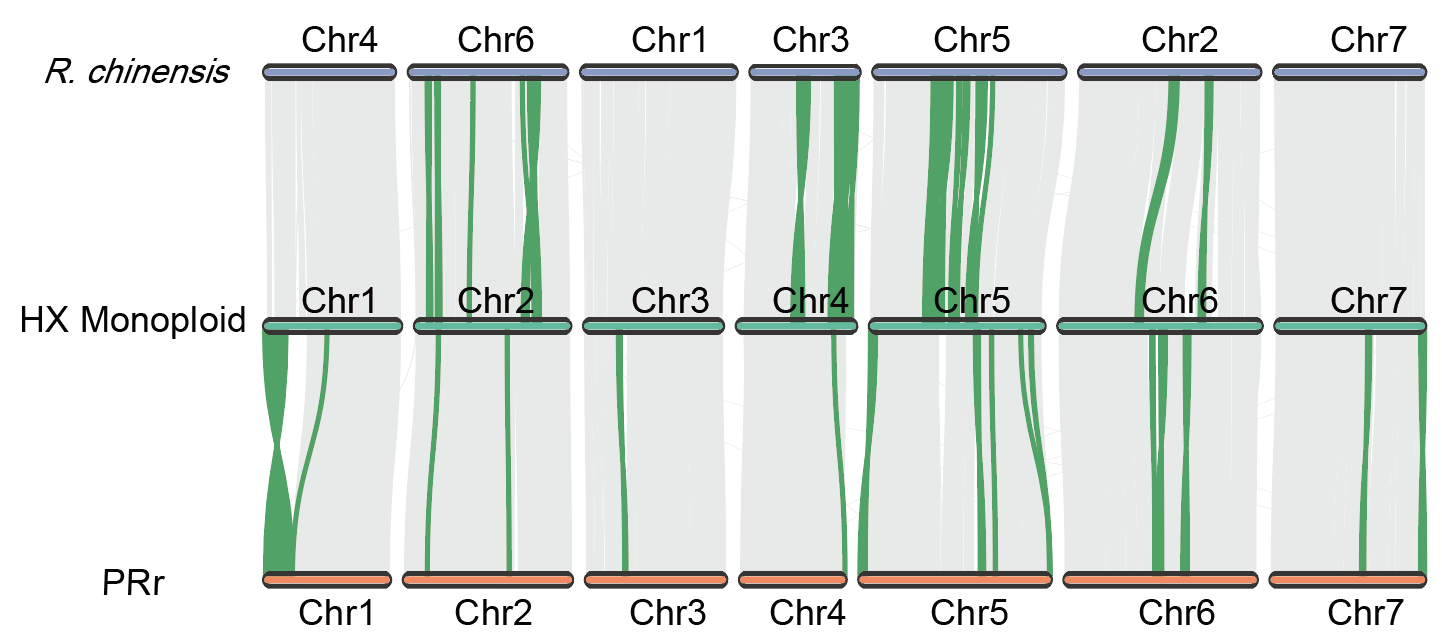


**Figure S9. Analysis of the synteny between the *R. chinensis* genome assembly, monoploid (HX) genome assembly, and PRr genome assembly.**

Green blocks represent large structural variations among the three genomes.


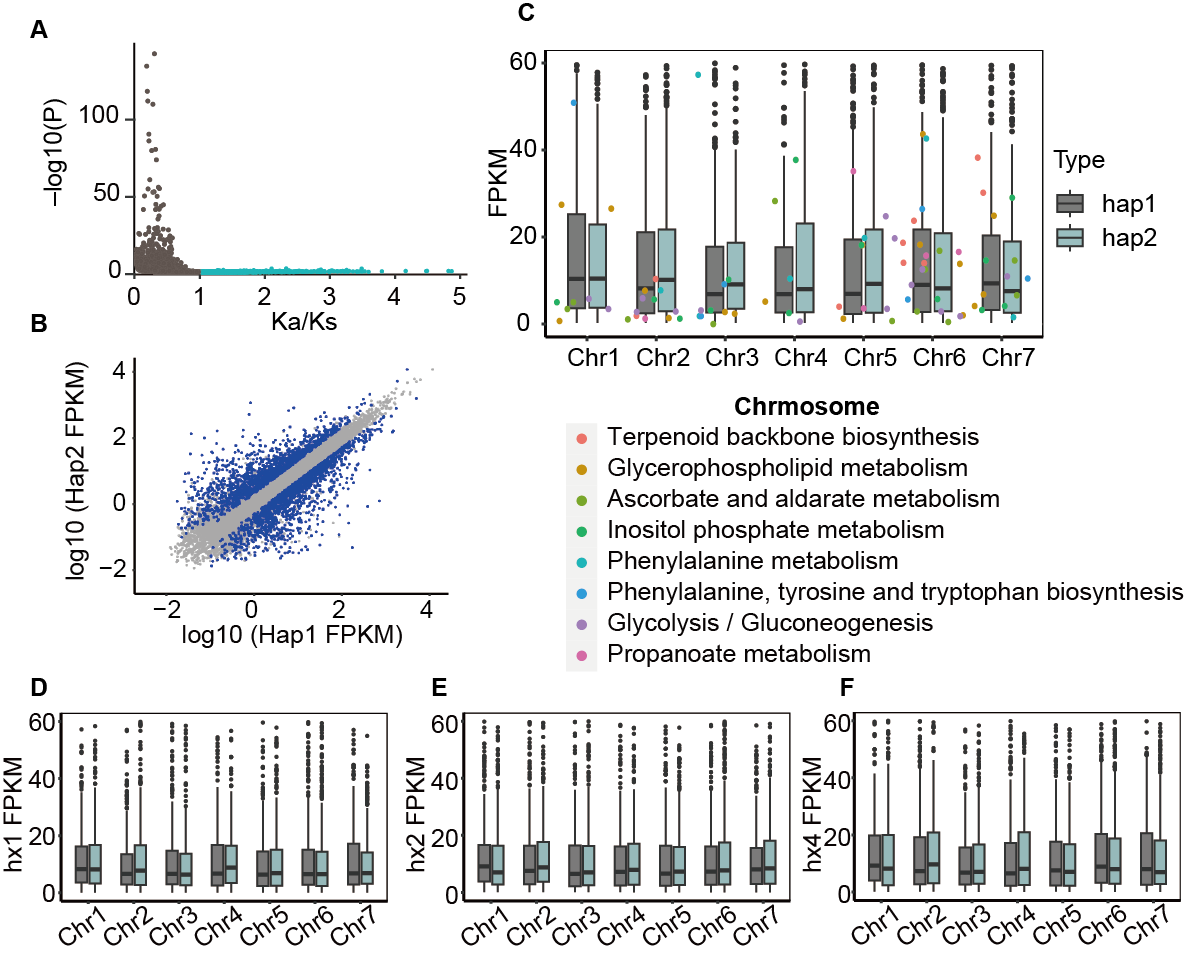


**Figure S10. The ASE gene’s expression in petals of hap1 and hap2 in bud stage (hx 1), initial opening period (hx 2), and fading period (hx 4) of HX.**

**(A)** Distribution of the *Ka*/*Ks* ratio among allelic genes. **(B)** dentification of ASE genes in leaves. Coordinates are logarithmically scaled (log_10_). Blue dots represent ASE genes, whereas gray dots represent non-ASE genes. FPKM, fragments per kilobase exon per million fragments mapped; Hap1, haplotype 1; Hap2, haplotype 2. The ASE gene’s expression in petals of hap1 and hap2 in **(D)** bud stage (hx 1), **(E)** initial opening period (hx 2), **(C)** bloom period (hx 3) and **(F)** fading period (hx4) of HX.


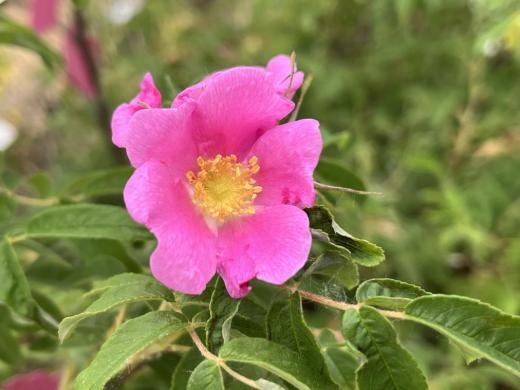

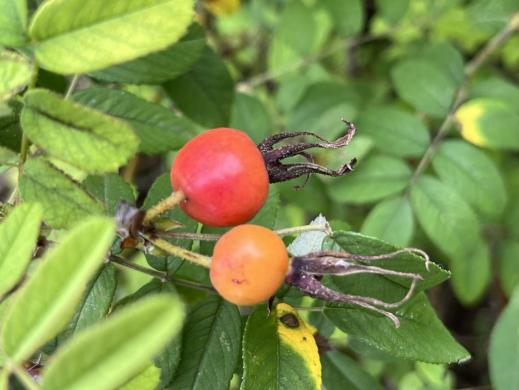


**Figure S11. Images of *R. rugosa* GM plant parts.**


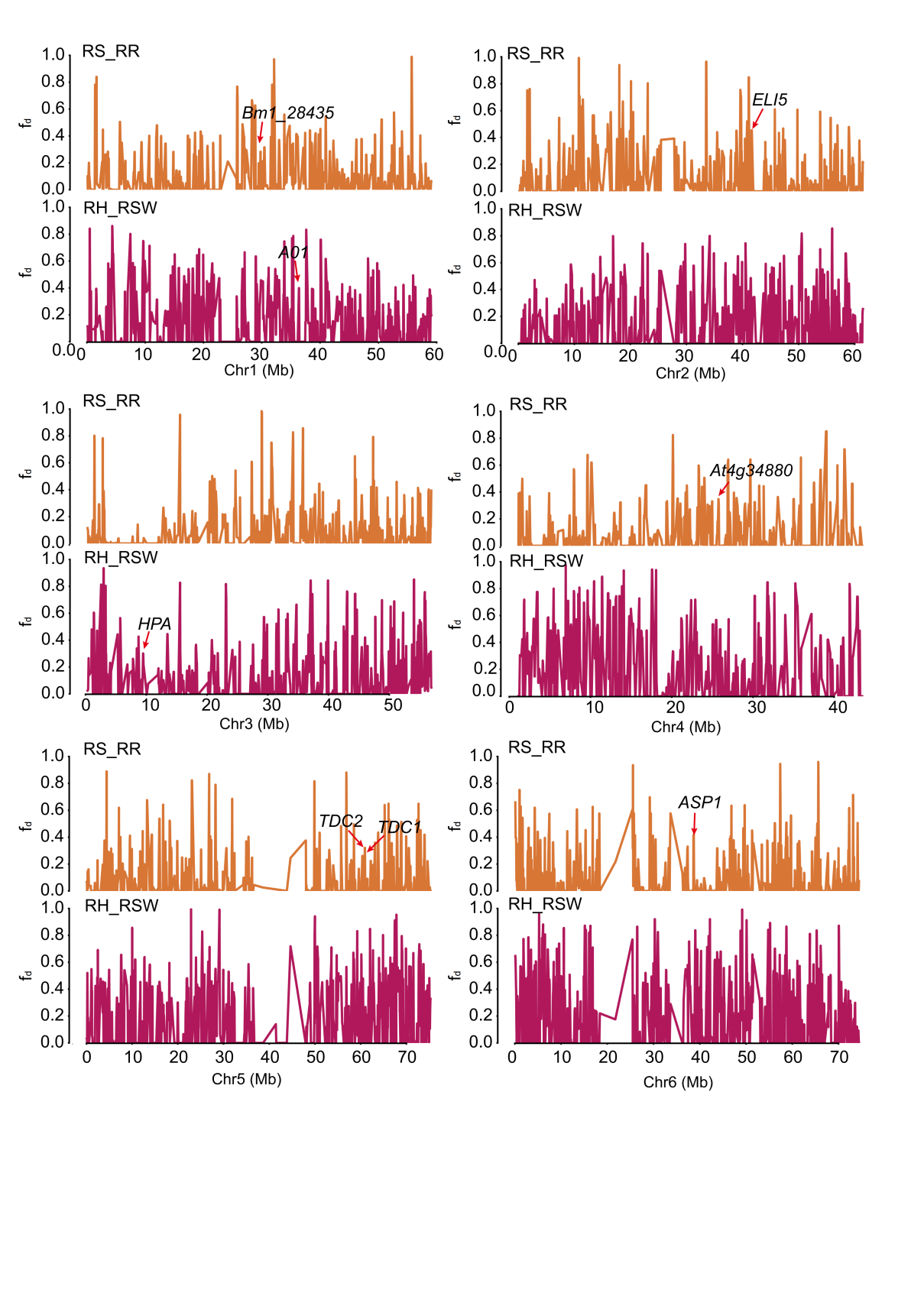


**Figure S12. Introgression from donor populations to acceptor across chromosome 1-6 (acceptor_donor).**


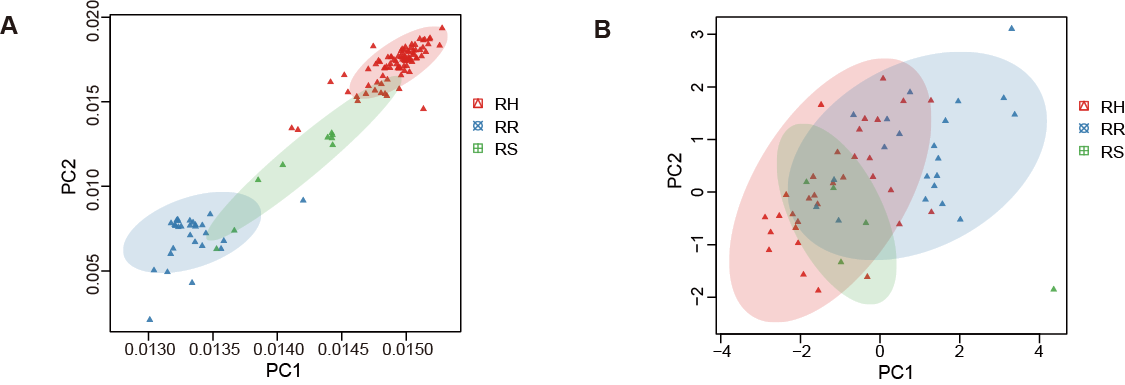


**Figure S13. The Principal Component Analysis of RR, RH and RS.**

PCA analysis with SNP **(A)** and components of rose scent **(B)** of RR, RH and RS.


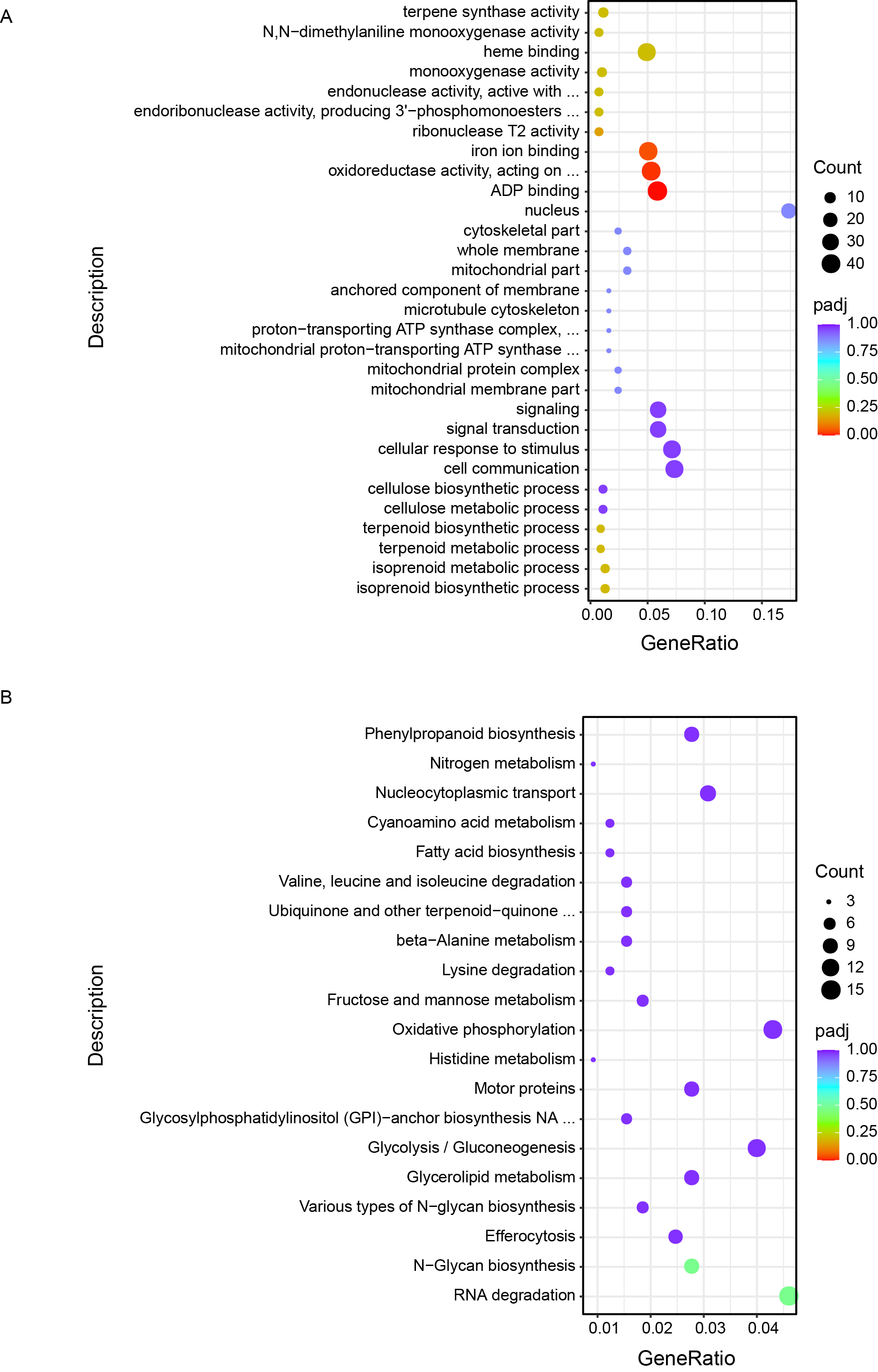


**Figure S14. Selected genes’ enrichment results.**

**(A)** GO enrichment of RS’s pi selected genes from comparison of RH and RS. **(B)** KEGG enrichment of *Fst* selected genes from RH vs RS.


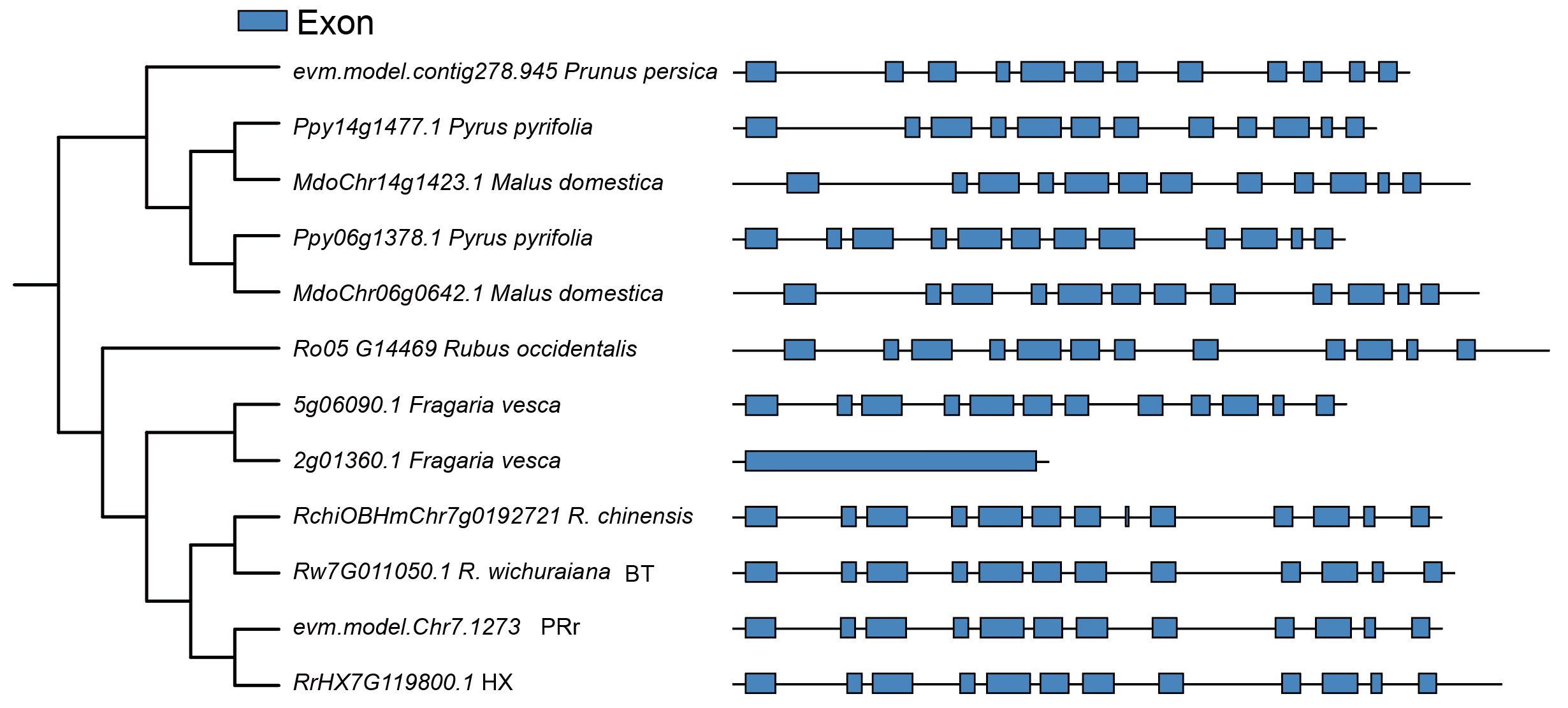
**Figure S15. Evolutionary tree of *RrHX7G119800* and gene structure.**

**Figure S16. *RrHX7G119800* homologous gene sequence comparison.**


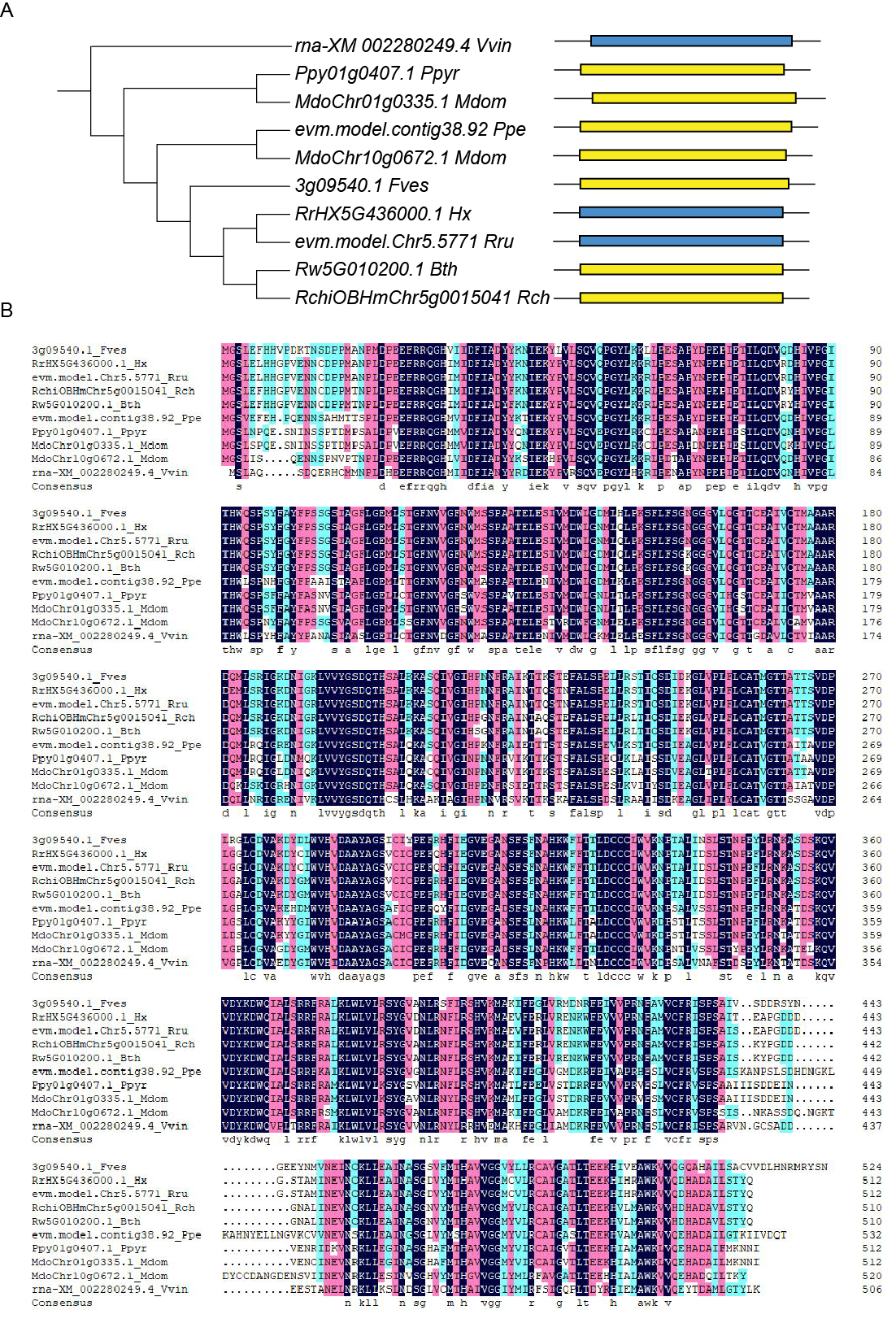


**Figure S17. A comparative analysis of RrTDC-encoding gene *RrHX5G43600*.**

**(A)** Evolutionary tree of *RrHX5G43600* and gene structure. **(B)** *RrHX5G43600* homologous gene sequence comparison.


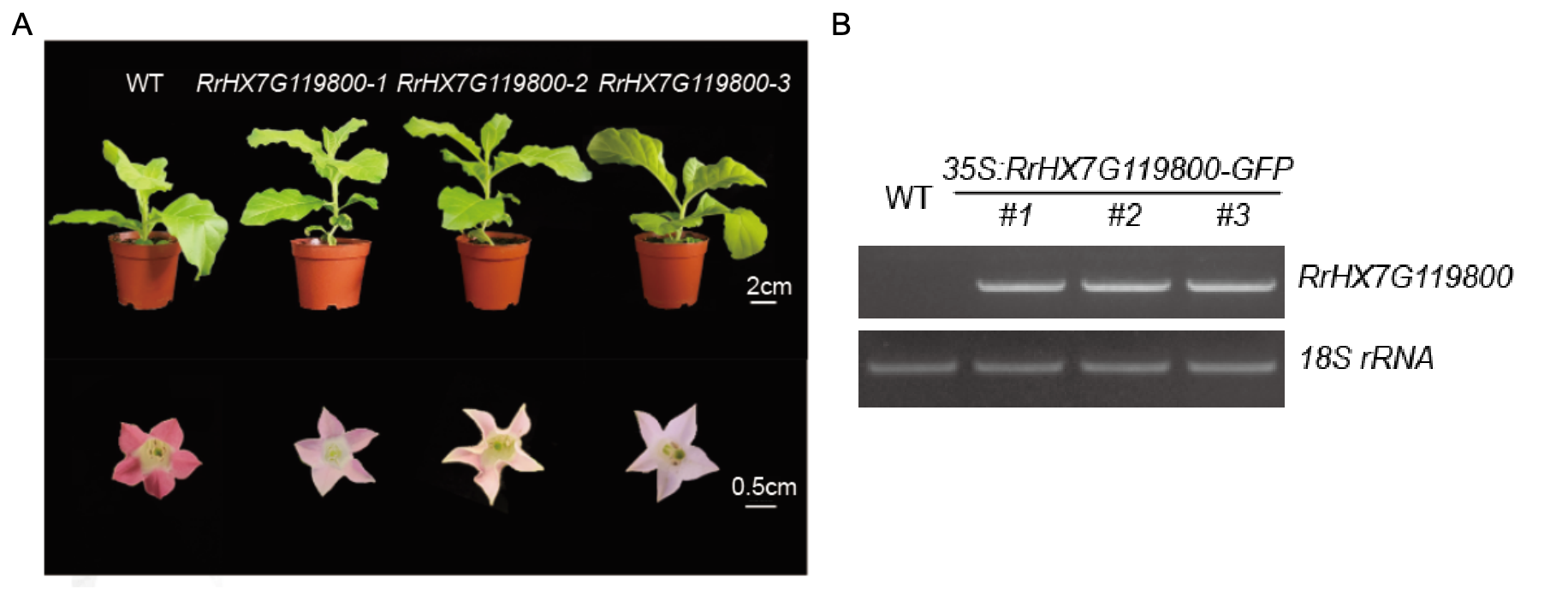


**Figure S18. Overexpressing *RrHX7G119800* in transgenic tobacco plants.** **(A)** Plants growth status of transgenic tobacco plants overexpressing three *RrHX7G119800* and WT. **(B)** RT-PCR revealed that *RrHX1G119800* expression levels in transgenic tobacco plants overexpressing *RrHX7G119800* and WT.
